# Scalable Fabrication of Metallic Nanogaps at the Sub‐10 nm Level

**DOI:** 10.1002/advs.202102756

**Published:** 2021-10-31

**Authors:** Sihai Luo, Bård H. Hoff, Stefan A. Maier, John C. de Mello

**Affiliations:** ^1^ Department of Chemistry Norwegian University of Science and Technology (NTNU) Trondheim NO‐7491 Norway; ^2^ Nano‐Institute Munich Faculty of Physics Ludwig‐Maximilians‐Universität München München 80539 Germany; ^3^ Blackett Laboratory Department of Physics Imperial College London London SW7 2AZ UK

**Keywords:** nanogap, nanofabrication, nanoelectronics, nanophotonics, plasmonics

## Abstract

Metallic nanogaps with metal–metal separations of less than 10 nm have many applications in nanoscale photonics and electronics. However, their fabrication remains a considerable challenge, especially for applications that require patterning of nanoscale features over macroscopic length‐scales. Here, some of the most promising techniques for nanogap fabrication are evaluated, covering established technologies such as photolithography, electron‐beam lithography (EBL), and focused ion beam (FIB) milling, plus a number of newer methods that use novel electrochemical and mechanical means to effect the patterning. The physical principles behind each method are reviewed and their strengths and limitations for nanogap patterning in terms of resolution, fidelity, speed, ease of implementation, versatility, and scalability to large substrate sizes are discussed.

## Graphical abstract

Metallic nanogaps with metal–metal separations below 10 nm have many applications in nanoscale photonics and electronics. However, their fabrication remains a considerable challenge, especially for applications that require patterning over macroscopic length‐scales. Here, some of the most promising fabrication methods are evaluated, with an emphasis on scalable techniques that can be used to rapidly pattern large‐area nanogap arrays.

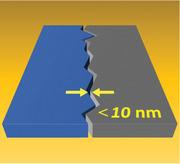

## 1 Introduction

In‐plane metal electrodes, separated on the nanometer length scale, are essential elements of many nanoscale photonic and electronic devices.^[^ ^1^, ^2^, ^3^ ^]^ The small gap widths make them ideal choices for all‐electronic biosensors since the capture of a single biomolecule within or across the metallic nanogap (MNG) can induce large measurable changes in the electrical characteristics.^[^ ^4^ ^]^ MNGs are essential components of molecular electronic devices, where conductive molecules are attached across the gap (individually or in groups) and serve as functional semiconductors in highly miniaturized switches,^[^ ^5^, ^6^, ^7^, ^8^ ^]^ rectifiers,^[^ ^9^, ^10^, ^11^ ^]^ or transistors.^[^ ^1^, ^12^ ^]^ MNGs also permit the manipulation of light via plasmonic interactions, with illumination of the nanogap inducing resonant oscillations of the free electrons inside the metal electrodes (surface plasmons).^[^ ^13^, ^14^, ^15^, ^16^ ^]^ The oscillating electrons act as electric dipoles that re‐emit light coherently at the same frequency as the incident radiation, while channeling a significant fraction of electromagnetic energy from the far field to highly confined near‐field regions within the nanogaps (i.e., they “concentrate” light inside the gap region). These optical near fields can be many orders of magnitude greater than the incoming light, allowing the nanogaps to act as highly localized sources of light, heat or energetic electrons for, for example, photocatalysis,^[^ ^17^, ^18^ ^]^ surface‐enhanced spectroscopy,^[^ ^19^, ^20^, ^21^ ^]^ terahertz optics,^[^ ^22^ ^]^ lasing,^[^ ^23^, ^24^ ^]^ photovoltaics,^[^ ^25^ ^]^ magnetoplasmonics,^[^ ^26^, ^27^ ^]^ and plasmonic circuits.^[^ ^28^ ^]^

For many applications, a gap‐width of less than 10 nm is required to achieve the desired functionality: for an MNG‐based device or sensor, it is typically necessary for the gap‐width to be smaller than the length of the molecular semiconductor or target biomolecule, that is, a few nanometers; while, for plasmonic applications, smaller gap widths give rise to higher optical field strengths, leading to stronger plasmonic effects.

This article is specifically focused on fabrication methods for MNGs. Useful review articles describing their many applications may be found elsewhere.^[^ ^1^, ^2^, ^12^, ^14^, ^29^ ^]^ In this paper, we describe a range of techniques capable of yielding nanogaps between metals, focusing in particular on methods that can both yield sub‐10 nm gap widths and be applied over large (application‐relevant) areas of several square millimeters. The different techniques vary in their resolution, speed, scalability, cost, materials compatibility, ease of implementation, and versatility, and no single method is suitable for all nanogap applications. We begin by reviewing several established technologies that have been successfully applied to the patterning of narrow metallic nanogaps: extreme UV (EUV) lithography, EUV‐ and UV/Vis‐interference lithography, e‐beam lithography (EBL), and focused ion‐beam (FIB) lithography. We then turn to a number of alternative techniques that use mechanical or electrochemical means to produce nanogaps—often at higher speed and lower cost than conventional methods—and point out some of the challenges that must be addressed before these newer methods are ready for widespread use.

## 2 Established Fabrication Methods

We begin by reviewing the three most established methods for nanofabrication: photolithography, EBL, and FIB lithography. The first uses photons to effect patterning, while the other two use beams of charged particles.

### 2.1 Photon‐Based Methods

**Figure 1:**
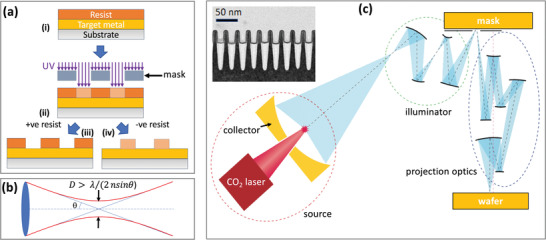
a) Schematic of shadow‐mask photolithography, in which i) a substrate is coated with a target metal and a photoresist, ii) the resist is selectively exposed with UV light via a photomask to modify the solubility of the resist, and iii,iv) the entire stack is immersed in a developer to remove the soluble parts of the resist. In the case of an (initially insoluble) positive resist, the exposed parts are rendered soluble (iii), while in the case of an (initially soluble) negative resist, the exposed parts are rendered insoluble. The exposed metal may be subsequently removed by chemical etching prior to removal of the resist. b) The smallest spot size *D* to which a point source may be focused by a mirror or lens is ∼*λ*/(2*n*sin*θ*), where *λ* is the wavelength of the illuminating light, *n* is the index of refraction of the surrounding medium, and *θ* is the half‐angle of the light cone entering the optic. c) Schematic of an Extreme‐UV projection lithography (EUV‐PL) system showing a laser‐produced plasma source that generates light of wavelength 13.5 nm, a collector mirror that focuses the light, illuminator optics that direct the light onto a reflective mask, and projection optics that redirect the light reflected by the mask to a wafer. The entire system operates in vacuum.^[^ ^36^ ^]^ Inset of (c) shows 7‐nm transistors with a 30 nm pitch fabricated by EUV projection lithography. c) Main image: Reproduced with permission.^[^ ^36^ ^]^ Copyright 2010, Springer Nature. Inset image is courtesy of IBM.

Photon‐based methods typically rely on the use of a thin layer of a light‐sensitive polymer (“photoresist”) to create three‐dimensional solid features that can then be used to transfer a pattern to a target material, see, for example, **Figure** **1**a. The pattern in the photoresist is typically induced by exposure to UV light, using, for example, a photomask or a scanning laser beam (“direct laser writing”, DLW). Under illumination, a photo‐acid generator inside the photoresist induces a chemical reaction that changes the local solubility. In the case of an initially insoluble positive resist, the exposed regions of the polymer are rendered soluble by acid‐catalyzed chain scission, while in the case of an initially soluble negative resist, the exposed regions of the polymer are rendered insoluble by acid‐catalyzed cross‐linking.^[^ ^30^ ^]^ The soluble parts of the resist are then removed by immersion in a “developer,” leaving behind a suitably patterned layer. The resist can be deposited directly onto a substrate and used as a stencil for shadow‐mask deposition of a metal; alternatively, it can be placed above a pre‐deposited metal (as shown in Figure 1a) and used to protect the underlying parts of the metal during chemical etching. When the resist is physically or chemically stripped away from the sample, a patterned metal is left behind, which is either similar to (etching) or the inverse of (shadow‐mask deposition) the photoresist pattern.

Photon‐based methods are the “work‐horses” of the semiconductor processing industry due to their high reliability and relative ease of implementation. However, they have not been widely used for patterning nanogaps at the sub‐10 nm level due to the fundamental resolution limits imposed by the wave nature of light. From Abbe's theory of diffraction,^[^ ^31^, ^32^ ^]^ the smallest spot size to which a point source may be focused by a mirror or lens is ≈*λ*/2*N*, where *λ* is the wavelength of the illuminating light and *N* is the numerical aperture of the focusing optic.^[^ ^15^, ^31^, ^33^, ^34^ ^]^ The numerical aperture is given by the equation *N*  =  *n*sin*θ* where *n* is the index of refraction of the surrounding medium and *θ* is the (maximal) half‐angle of the light cone entering the optic, see Figure 1b. For a dry lens or mirror (in air or vacuum) *N* cannot be greater than one (the refractive index of free space), meaning the spot size can be no smaller than half the wavelength of the illuminating light, that is, a few hundred nanometers for visible light. A moderate (≈40%) reduction in spot size (relative to that obtained using a dry lens) may be achieved by flooding the space between the focusing lens and the illuminated surface with a liquid that has a refractive index greater than one—a process known as immersion lithography.^[^ ^35^ ^]^ Modern immersion lithography systems using a 193 nm ArF laser and ultrapure water as the liquid medium (*n*  = 1.44 at 193 nm) are capable of producing optical spot sizes smaller than 70 nm.^[^ ^36^ ^]^

To achieve patterning at the few nanometer length‐scale, it is typically necessary to use extreme UV (EUV) radiation.^[^ ^34^, ^36^ ^]^ In EUV projection lithography (EUV‐PL), a uniform beam of 13.5 nm light from an intense EUV source is directed toward a patterned, reflective binary photomask, and the reflected light is then de‐magnified and projected onto a resist‐coated wafer (see Figure 1c). EUV‐PL is the method of choice for ultrahigh resolution semiconductor manufacturing in terms of speed, accessible feature size and shape‐versatility. However, the technology presents major technical challenges and has taken almost forty years to reach commercial maturity. In particular, EUV light is both difficult to generate and strongly absorbed by virtually all media (including air), which prevents the use of conventional transmissive optics. In a typical EUV‐PL system, the condenser optics for the light‐source, the projection optics, and the photomask are all based on Distributed Bragg Reflectors (DBRs) formed from alternating layers of molybdenum and silicon (with thicknesses tuned to maximize reflectivity at 13.5 nm). Production tolerances are extremely tight: mirrors must have a surface flatness of less than 2 nm over a typical diameter of 30 cm, while masks must be virtually defect‐free to avoid patterning errors. Moreover, since reflective losses at each component are ≈60–70%, only a small percentage of the light collected from the source reaches the photoresist,^[^ ^34^ ^]^ and extremely bright light‐sources (typically based on a laser‐induced liquid tin discharge plasma) must therefore be used.^[^ ^34^, ^37^ ^]^ These factors (and many others) make EUV‐PL an extremely costly technology that is practical only for state‐of‐the‐art semiconductor manufacturing, where it is currently being used to pattern feature sizes below 10 nm. At the time of writing, EUV‐PL systems are produced by only one company (ASML) at a typical cost of US$100m, while low defect mask blanks cost more than $100k.

**Figure 2:**
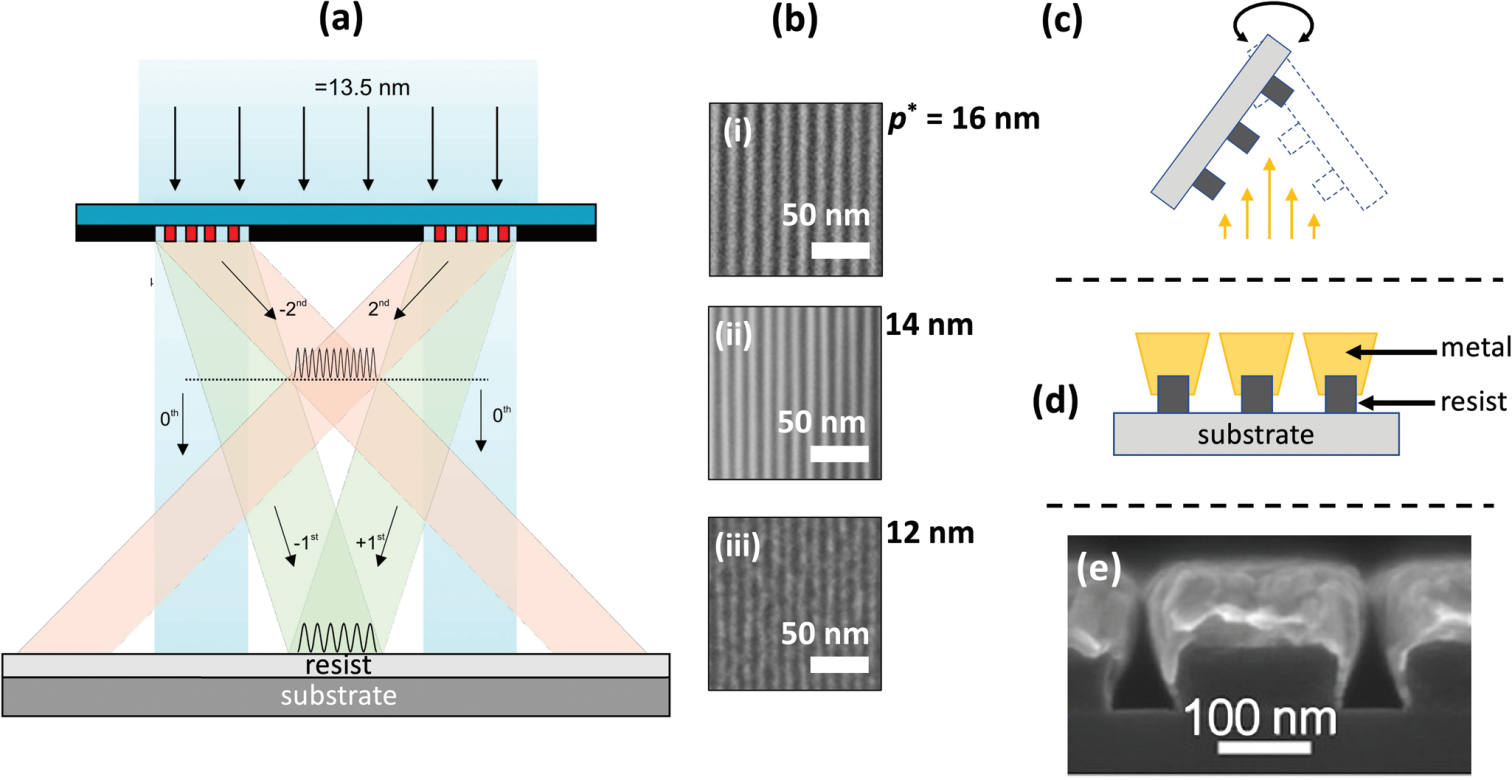
a) Schematic of extreme UV interference lithography (EUV‐IL), in which coherent light from an EUV light‐source strikes two closely spaced gratings, resulting in interference fringes where the diffracted beams overlap. The periodicity of the first‐ and second‐order fringe patterns are equal to one‐half and one‐quarter the grating periodicity, respectively.^[^ ^38^ ^]^ b) SEM images of line arrays with pitches of i) 16 nm, ii) 14 nm, and 12 nm, obtained by EUV‐IL using respective grating pitches of 64, 56, and 48 nm, and second‐order overlap.^[^ ^39^ ^]^ c) Schematic showing glancing‐angle metal deposition onto an EUV‐IL patterned line‐array of HSQ resist. d) Schematic showing appearance of line‐array after glancing‐angle deposition. e) SEM image showing nanogap line‐array obtained by glancing‐angle metal deposition, using an EUV‐IL patterned line‐array with an initial pitch of 250 nm. After metal deposition, the gap‐size has been reduced to approximately 12 nm.^[^ ^40^ ^]^ a) Reproduced with permission.^[^ ^38^ ^]^ Copyright 2011, IOP Publishing Ltd. b) Reproduced with permission.^[^ ^39^ ^]^ Copyright 2016, Proceedings of SPIE. e) Reproduced with permission.^[^ ^40^ ^]^ Copyright 2011, American Institute of Physics.

For research purposes, an alternative—less costly—EUV‐based method known as EUV interference lithography (EUV‐IL) may be used to pattern high resolution nanostructures (see **Figure** **2**a). In EUV‐IL spatially coherent EUV light derived from a synchrotron, laser or plasma source strikes two adjacent gratings of equal period *p*_0_, generating a series of diffracted beams that are oriented at discrete angles *θ*_*m*_ to the grating normal. For normally incident monochromatic light of wavelength *λ*, the permitted values of *θ*_*m*_ are determined by the grating equation *p*_0_sin *θ*_*m*_ =  *mλ* where *m* is a small integer that denotes the order of diffraction. Interference fringes are formed where diffracted beams overlap. For two overlapping beams of equal order m*, the periodicity p* of the fringes is given by p* = *p*_0_ /2m*. Hence, if a sample is placed in the overlap region of the second‐order beams (m* =  2), a fourfold decrease in feature size can be achieved relative to the gratings.^[^ ^38^ ^]^ In practice, for ultrahigh resolution patterning, the minimum attainable feature size is typically limited by the performance of the EUV resist. In recent work Buitrago and co‐workers reported a negative tone resist based on tin oxide and hafnium oxide that was capable of yielding high quality line arrays with periodicities down to 14 nm (see Figure 2b).^[^ ^39^ ^]^ Although not reported by the authors, the resist pattern should in principle be transferrable to a metal by etching or shadow‐mask deposition.

In earlier work using lower resolution gratings, EUV‐IL was successfully combined with glancing angle thermal evaporation to produce sub‐10 nm nanogaps.^[^ ^40^, ^41^ ^]^ In this approach, EUV‐IL was first used to pattern a large‐area (1 mm^2^) line array of 250‐nm periodicity in a 80‐nm layer of hydrogen silsesquioxane (HSQ) resist. Glancing angle thermal evaporation was then used to incrementally coat the resist with chromium in 4‐nm steps, rotating the substrate 180 degrees between each 4‐nm deposition to achieve a balanced coating on opposing sides of the lines (see Figure 2c,d). The minimum gap‐width between the metal‐coated lines decreased steadily as the thickness of deposited metal increased, reaching values as low as 5 nm (Figure 2e). However, it should be noted that the nanogaps were formed by glancing angle thermal evaporation onto relatively coarse gratings, and other methods could in principle have been used to fabricate the gratings, for example, deep UV lithography using 193 nm light from an ArF excimer laser.

EUV‐IL is capable of rapidly patterning very small features over large areas, but is limited by the high cost and scarcity of suitable light sources, the need to pattern the diffraction gratings by other methods such as EBL, and severe limitations in the range of feature shapes that can be patterned. As an alternative to using EUV radiation, a number of other photon‐based methods have been developed to circumvent the diffraction limit.^[^ ^33^, ^42^, ^43^, ^44^, ^45^ ^]^ Most of these techniques rely on the observation that Abbe's limit applies only to the minimum spot size of the illuminating radiation and not to the size of the photo‐induced features.^[^ ^32^ ^]^ The photo‐induced features are strongly influenced by the chemical properties of the resist, and hence they can be bigger or smaller than the Abbe spot size.

In the case of a line‐array formed by two‐beam interference lithography, the shortest resolvable periodicity is strictly limited by Abbe's theorem and can therefore be no smaller than *λ*/2*N*, but the individual lines within the line‐array can in principle be made arbitrarily thin by modifying both the chemical nature and the handling of the resist.

**Figure 3:**
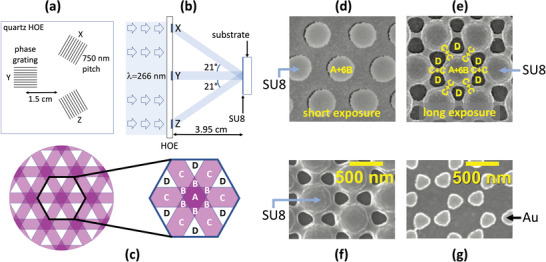
a) Schematic of Holographic Optical Element (HOE) used for three‐beam interference lithography,^[^ ^47^ ^]^ comprising a 6‐cm × 6‐cm quartz slide with three etched phase‐gratings (*X*, *Y*, and *Z*) arranged at 120° to one another. b) Schematic showing experimental set‐up for three‐beam interference lithography. The HOE is illuminated with an expanded 266‐nm laser beam, and an SU8‐coated substrate is placed a distance of 3.95 cm from the HOE where the three first‐order diffraction beams overlap, generating an interference pattern with hexagonal symmetry. c) Schematic of the three‐beam interference pattern, in which zones A, B, C, and D correspond to regions of high, medium, low, and very low intensity according to the degree of constructive or destructive overlap between the beams. d) Typical scanning electron micrograph showing the SU8 pattern due to a short exposure time, in which cross‐linking occurs only in the highest intensity zones A and B, generating isolated, cylindrical rods of SU8. e) Typical scanning electron micrograph showing the SU8 pattern due to a longer exposure time, in which additional cross‐linking occurs in zone C, generating bridges between the cylindrical rods; weakly exposed SU8 in zone D is removed during developing, creating holes on each side of the bridges. f,g) Scanning electron micrographs showing a typical shadow‐mask template obtained by three‐beam interference lithography and the corresponding gold nanoarray. Adapted with permission.^[^ ^47^ ^]^ Copyright 2020, Royal Society of Chemistry.

Three‐ and four‐beam interference lithography allows for the generation of two dimensional periodic patterns.^[^ ^46^, ^47^, ^48^ ^]^ The periodicity of the interference patterns is still governed by Abbe's theorem and hence cannot be reduced much below half the wavelength of the illuminating radiation, but nanoscale features within the repeating motif of the interference pattern can be used to define nanogaps. Liu et al. for instance used a holographic optical element (HOE) to generate a three‐beam interference pattern.^[^ ^47^ ^]^ The HOE was formed by lithographically patterning a 6‐cm × 6‐cm quartz slide with three 1‐cm × 1‐cm linear phase‐gratings of period 750 nm and depth 300 nm (**Figure** **3**a). The three gratings were positioned equal distances (1.5 cm) from the center of the quartz slide and oriented at 120° to each other. When illuminated (at normal incidence) by an expanded 266‐nm laser‐beam, the three transmissive gratings generated square‐shaped first‐ and second‐order beams at diffraction angles of 21° and 45°, respectively. An SU8‐coated substrate was placed a perpendicular distance of 3.95 cm from the HOE in the plane where the first‐order beams overlapped (Figure 3b), resulting in a large‐area interference pattern of broadly equal size to the transmission gratings (1 cm × 1 cm).

The interference pattern is shown schematically in Figure 3c. The authors identified four zones A, B, C, and D corresponding to regions of high, medium, low, and very low intensity according to the degree of constructive or destructive interference. For short exposure times, only the most heavily exposed zones (A and B) underwent cross‐linking, giving rise to a hexagonal array of cylindrical rods when the SU8 was subsequently developed (Figure 3d). Longer exposure times led to cross‐linking in other less strongly exposed parts of the SU8, causing a broadening of the cylindrical rods and the formation of bridges between the rods due to cross‐linking in zone C (Figure 3e). Zone D remained unexposed, giving rise to holes either side of the bridges when the SU8 was subsequently developed. The SU8 pattern was subsequently transferred to a gold layer via a combination of shadow‐mask deposition and lift‐off lithography, with the gold being deposited in the location of the holes (Figure 3f,g). The deposited gold formed a hexagonal array of doublets described by the authors as “nano‐bowties.” The separation between the two halves of each bowtie was determined by the width of the SU8 bridges, which in turn was determined by the exposure time (with shorter exposure times yielding narrower bridges). For the shortest reported exposure time of 5 s an SU8 bridge‐width of 25 nm was obtained, which translated to a gold‐to‐gold separation of just 13 nm in the final metallic array.

**Figure 4:**
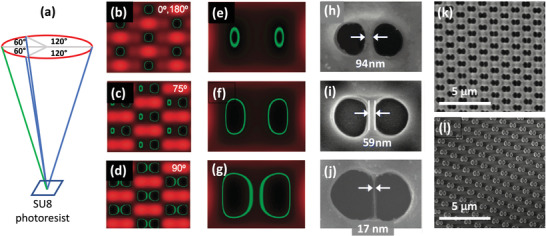
a) Schematic showing experimental set‐up for four‐beam interference lithography, in which three of the beams are oriented at 120° to one another (when projected onto the sample plane), while the fourth beam is oriented at 180° to one of the other three beams.^[^ ^48^ ^]^ b–d) Simulated interference patterns showing the effects of introducing a phase‐delay of 0°, 75°, or 90° into one of the four laser beams. The regions inside the green contours correspond to areas where the intensity is too low to induce cross‐linking of SU8, and hence holes are expected to form when the resist is developed. For zero phase‐delay, a hexagonal array of equally sized singlet holes is expected; while, for a 90° phase‐delay, a hexagonal array of equally sized doublet holes is expected. e–g) Simulated interference patterns showing—for a fixed phase‐delay of 90° in one of the laser beams—an increase in the size of the low intensity regions as the beam intensity is increased from low (e) to high (g). h–j) Scanning electron micrographs showing—for a fixed phase‐delay of 90° in one of the beams—the formation of doublet holes of increasing size and decreasing separation as the beam intensity is increased from low (h) to high (j). At the highest intensity an average hole separation of ≈20 nm is obtained. The SEM images are in broad agreement with the simulated results shown in (e–g). k,l) Low magnification scanning electron micrographs showing a typical shadow‐mask template (k) obtained by four‐beam interference lithography with a phase‐delay of 90° in one of the beams, and the corresponding gold nanoarray (g). Adapted with permission.^[^ ^48^ ^]^ Copyright 2011, American Chemical Society.

Zhang et al. used four‐beam interference lithography at a (visible) wavelength of 488 nm to generate large‐area nanogap arrays with gap‐sizes that were far below the wavelength of the illuminating radiation.^[^ ^48^ ^]^ The exposure pattern was generated by splitting a laser beam from an argon ion laser into four daughter beams of equal intensity, and recombining the beams on a substrate coated with the negative photoresist SU8 (**Figure** **4**a). Three of the beams (shown in blue in Figure 4a) were oriented at 120° to one another (when projected onto the sample plane), while the fourth beam (shown in green) was oriented at 180° to one of the other three beams. This beam arrangement was chosen to give a hexagonal interference pattern, in which the periodicity of the interference pattern could be controlled by changing the incident angle of the laser beams, and the structure of the repeating motif could be tuned by varying the relative intensities, phases and polarization axes of the daughter beams.

Figure 4b–d shows how the (simulated) interference pattern due to four p‐polarized laser beams of equal intensity is changed when a phase delay is introduced into one of the laser beams. The green contour lines enclose the lowest intensity regions where no crosslinking occurs and SU8 is therefore lost during the developing stage. With a phase‐delay of zero (Figure 4b), a hexagonal array of singlet circular holes is expected. Increasing the phase‐delay splits the singlet holes into doublet holes, with the relative size of the two holes depending on the value of the phase‐delay. At a phase delay of 90° (Figure 4d), the doublet holes are equally sized and serve as a convenient deposition mask for defining symmetric bowtie‐shaped metallic features.

Figure 4e–g shows the effect on the interference pattern of changing the intensity of the parent beam when there is a fixed 90° phase‐delay in one of the daughter beams, giving rise to symmetric doublet holes. The low intensity regions grow in size as the beam intensity is increased, which leads to larger and more closely spaced doublet holes in the SU8. The authors reported that the average hole separation within each doublet could be tuned from ≈100 nm to <20 nm by increasing the intensity of the parent beam (Figure 4h–j), with the two holes merging into a single large hole at very high beam intensities. A low magnification SEM image of an SU8 nanohole array is shown in Figure 4k. Using the patterned SU8 as a contact shadow‐mask, bowtie‐shaped metallic nanogap arrays with a mean separation of 22 nm and a minimum gap width of 7 nm were obtained (Figure 4l). The total area was determined by the 2500 µm^2^ overlap area of the interfering beams, and in principle could have been scaled to larger areas by expanding the parent laser beam (as for instance was done in the work of Liu et al.^[^ ^47^ ^]^).

Overall, multibeam interference lithography using UV or visible light is a rapid, reliable, scalable, and cost‐effective method for fabricating metallic nanogap arrays with gap‐widths of a few tens of nanometers. To our knowledge, arrays of ultranarrow nanogaps with mean gap‐widths below 10 nm have not yet been demonstrated, but it is reasonable to assume this would be possible with further process optimization. The principal drawback of multibeam interference lithography is the limited palette of motif shapes that can be achieved, and the need to exploit “accidental” nanoscale artefacts within the motif to achieve patterning at the low‐nm scale, which precludes the optimization of shape for specific applications.

For device applications where control of shape is essential, "multiple‐patterning" techniques offer a powerful and well‐established means of circumventing the diffraction limit. In multiple‐patterning,^[^ ^36^ ^]^ a dense pattern is split across several masks such that the feature spacing on each mask is larger than Abbe's limit, while the individual features themselves are substantially smaller than Abbe's limit. For instance, single‐step patterning by 193 nm immersion lithography cannot easily achieve line‐array periodicities better than about 80 nm. However, by using double patterning with a 40‐nm spatial offset between the two lithographic steps, line array periodicities can be reduced to around 40 nm.^[^ ^49^ ^]^ By using even more lithography steps, the resolution can be improved still further. Multiple patterning neatly gets around the limitations imposed by Abbe's limit at the expense of additional processing complexity. In particular, it increases the number of lithography steps needed and demands extremely high overlay registration between each one of those steps, which adds considerable complexity and cost to the approach. A number of so‐called self‐aligned methods have been developed in recent years to circumvent this challenge by avoiding the need for precise overlay registration.^[^ ^50^, ^51^ ^]^

**Figure 5:**
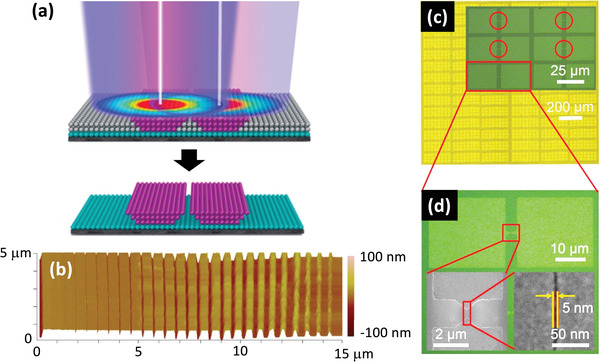
a) Illustration of nanogap fabrication using two closely spaced visible‐light laser beams and a thermally‐activated negative photo‐resist.^[^ ^45^ ^]^ The activation area due to each laser beam is smaller than the ≈200‐nm spot‐size since only resist that is located near to the center of the spot is heated sufficiently to undergo chemical conversion. By scanning the laser beams in a line and then immersing the sample in a developer to remove the unexposed parts of the resist, two parallel ridges of insoluble resist are generated. The width of the ridges—and hence the separation between them—is controlled by changing the laser intensity, with higher intensities yielding wider ridges with narrower separations. b) AFM image showing reduction in gap‐width in Ti/SiO_2_ bilayer from 200 nm (far left) to 18 nm (far right) as the laser intensity is increased from 40 to 80 mW. c) Top‐view optical images of a metallic nanogap electrode array, obtained using the thermally activated negative photo‐resist. d) Micrograph and SEM images of one nanogap within the array in (c). Reproduced with permission.^[^ ^45^ ^]^ Copyright 2020, American Chemical Society.

In some limited cases it is possible to engineer photoresists to produce very small feature sizes using direct laser writing at visible wavelengths. Using 405 nm DLW lithography, Qin and co‐workers found it was possible to achieve a tunable size‐mismatch between the illuminating radiation and the patterned feature size by using a negative inorganic resist that could only be activated above a specific thermal threshold.^[^ ^45^ ^]^ When the resist was exposed with a weak, Gaussian‐shaped laser beam at 405 nm, only the region at the center of the laser spot was heated sufficiently to undergo chemical conversion. For the lowest usable laser intensity, the diameter of the activation area was reduced to ≈60 nm compared to a diffraction‐limited spot size of >200 nm. At higher beam intensities, the activation area was broadened due to increased heating of the periphery areas away from the center of the spot. Hence, the conversion area could be straightforwardly controlled by varying the laser intensity. By scanning the beam in a line and then immersing the sample in a developer to remove the unexposed parts of the resist, a ridge of insoluble resist was generated, where the ridge‐width was determined by the beam intensity. Carrying out this procedure with two overlapping but slightly offset laser beams resulted in two parallel ridges separated by a small gap (**Figure** **5**a). Increasing the beam intensity caused the width of the ridges to increase and hence the width of the gap between them to decrease. Under conditions of constant pulse duration and spot‐size, the width of the region between the ridges could be decreased from 200 to 18 nm by increasing the laser power from 40 to 80 mW (Figure 5b). By carefully optimizing the beam intensities and scanning rates, the researchers were able to pattern nanogap arrays over macroscopic length‐scales with gap‐widths as small as 5 nm. Finally, a 15‐nm layer of gold was deposited on top of the patterned substrate by e‐beam evaporation, yielding a metallic nanogap of width ≈5 nm that was successfully used in the fabrication of arrays of molecular diodes (Figure 5c,d).

Other reported techniques for circumventing the diffraction limit include plasmonic lithography (where metal contact masks with regular arrays of sub‐wavelength holes or slits are used to achieve highly localized illumination of an adjacent photoresist)^[^ ^31^, ^52^, ^53^, ^54^ ^]^ and two‐photon lithography (where only the central region of a tightly focused laser beam is sufficiently intense to induce two‐photon‐mediated conversion of the photoresist, leading to a narrowing of the effective beam size).^[^ ^33^, ^42^, ^43^ ^]^ However, there have been only a few reports of these methods being successfully used to fabricate metallic nanogaps with spacings less than 10 nm.^[^ ^42^, ^54^ ^]^

### 2.2 Charged Particle‐Based Methods

Charged particle‐based methods are similar to direct laser writing, except they use a moveable source of electrons or ions instead of photons as the conversion‐inducing stimulus, making use of the short de Broglie wavelength of the particles to achieve higher resolution patterning.^[^ ^55^, ^56^, ^57^, ^58^, ^59^, ^60^, ^61^ ^]^ Compared to photons, charged particles can be accelerated to very high energies (i.e. very low wavelengths), and consequently they can be focused to spot sizes as small as 1 nm. In addition, beams of charged particles typically have a large depth of focus (several microns or more). Consequently, the spot size remains small for considerable distances above and below the focal plane, which allows for high‐resolution patterning even on tilted surfaces.^[^ ^62^, ^63^ ^]^

**Figure 6:**
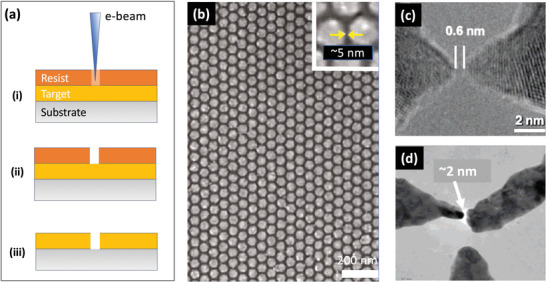
a) Schematic of EBL using a positive resist, in which i) the selected parts of the resist are rendered soluble by exposure to the scanning e‐beam, ii) the exposed resist is removed using a developer, iii) the exposed parts of the target layer are etched away, and the resist is removed, leaving a patterned layer of the target material. b) Low‐magnification SEM image showing gold nano‐disc arrays with ≈5 nm gaps, obtained using EBL and the negative photoresist HSQ.^[^ ^60^ ^]^ c,d) Two and three‐way electrodes separated by <2 nm fabricated by electron beam milling, that is, by using a high beam‐current electron beam to directly ablate atoms from c) a platinum target and d) a silver target.^[^ ^67^, ^68^ ^]^ b) Reproduced with permission.^[^ ^60^ ^]^ Copyright 2011, American Chemical Society. c) Reproduced with permission.^67^ Copyright 2007, American Chemical Society. d) Reproduced with permission.^[^ ^68^ ^]^ Copyright 2007, American Chemical Society.

The most widely used charged‐particle‐based method is electron beam lithography (see **Figure** **6**a), in which an electron‐sensitive resist is exposed with a beam of low‐to‐medium energy electrons (<100 keV) generated by a heated filament (normally tungsten or lanthanum boride) or a field emission gun (normally tungsten).^[^ ^64^ ^]^ For a fairly typical 30 keV electron source, the wavelength is around 7 pm—three orders of magnitude lower than the typical photon wavelength used in EUV lithography. In consequence, the resolution limits for EBL are not limited by the wavelength of the incident electrons; instead they are determined by factors such as the quality of the focusing optics, electron secondary scattering, and the chemical structure of the resist. In practice, the best achievable resolution for EBL is around 2 nm.

EBL is a reliable and well controlled process. However, it is slow compared to photon‐based lithography, with exposure times often exceeding 24 hours per square centimeter.^[^ ^65^ ^]^ Writing speeds can be elevated by increasing the beam current but this results in larger spot sizes and reduced patterning resolution due to increased electron repulsion. Hence, while there have been numerous reports of EBL‐fabricated MNGs, there are few reports of the technique being successfully used for large‐area fabrication. To minimize the electron beam writing time, the features written by the e‐beam should directly define the gap regions in the final pattern (since in most cases the nanogaps will typically account for only a small fraction of the total substrate area). Duan and co‐workers for instance used the negative resist hydrogen silsesquioxane (HSQ) to pattern features of approximate width 5 nm on a silicon substrate. Using the developed resist as a contact mask, metal was evaporated onto the substrate, and the resist was then removed in a conventional lift‐off process, leaving ≈5‐nm gaps where the resist had been exposed. Since only the gap regions were exposed during the e‐beam writing stage, arrays as large as 12 µm × 12 µm could be patterned with very low defect densities, see for example, Figure 6b.^[^ ^60^ ^]^

As an alternative to EBL, it is also possible to carry out electron beam milling (EBM), in which high‐energy electrons (>200 keV) are used to sputter atoms directly from a target film without the need for any resist layer.^[^ ^66^, ^67^, ^68^, ^69^, ^70^, ^71^ ^]^ Two and three terminal electrodes separated by <2 nm have been fabricated in this way (Figure 6c,d). However, the throughput was reported to be extremely low (several minutes per individual device) due to the slow rate at which atoms were sputtered by the incident electrons.^[^ ^67^ ^]^

Although EBL may be used to pattern sub‐10 nm features in a resist, it is not well suited to the patterning of high‐density arrays due to substantial scattering of the electrons by the substrate and the resist, which distorts the edge‐profile of the patterned features. The degree of distortion is affected by the presence of nearby patterned features in the resist; distortions arising from scattering events are consequently known as “proximity effects.” Compared to electron beams, ion beams are far less susceptible to scattering and so exhibit much weaker proximity effects, making them a better choice for high density nanoscale patterning.^[^ ^63^ ^]^

**Figure 7:**
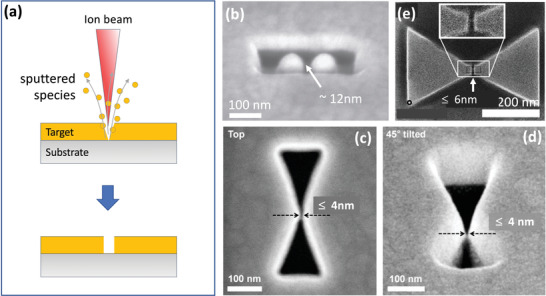
a) Schematic of FIB milling technique, where a nanogap is formed by using a scanning ion beam to directly sputter atoms from the target material. b) SEM image of a gold dimer antenna obtained by Ga FIB milling. The two gold islands at the center are separated by a ≈12 nm gap.^[^ ^73^ ^]^ c,d) Top‐ and tilted‐view SEM images of bowtie shaped air‐gaps in gold with ≈4‐nm minimum separation, obtained by Ga FIB milling.^[^ ^72^ ^]^ e) Example of a bow‐tie shaped gold dimer with a gap‐width of ≈6 nm obtained using a combination of Ga and He ion FIB milling for coarse‐ and fine‐resolution patterning, respectively.^[^ ^75^ ^]^ b) Reproduced with permission.^[^ ^73^ ^]^ Copyright 2013, Nature Publishing Group. c,d) Reproduced with permission.^[^ ^72^ ^]^ Copyright 2015, American Chemical Society. e) Reproduced with permission.^[^ ^75^ ^]^ Copyright 2014, American Chemical Society.

Patterning by ion beams is known as Focused Ion Beam milling and, like EBM, has the advantage of not requiring a resist (see **Figure** **7**a). In a typical set‐up, liquid gallium wets a positively biased tungsten needle, causing a beam of Ga^+^ ions to be ejected from the tip. The ions strike a target, causing atoms of the target to sputter from the surface. The best achievable resolution using Ga FIB milling is around 10 nm, which is set by the minimum achievable diameter of the ion beam (around 5 nm) and interactions between the ions and atoms in the target that blur the pattern.^[^ ^3^, ^72^ ^]^ FIB milling is much faster than electron‐beam milling but, since it causes some ions to be incorporated into the target material, it leads to unavoidable changes in chemical composition and crystallinity. Figure 7b shows gold dimer antennae with a ≈12 nm gap, which is close to the best resolution that can be achieved by standard Ga FIB milling.^[^ ^73^ ^]^ However, it is possible to improve the resolution somewhat by deliberately modifying the scan path of the beam to correct for interactions between the ions and the atoms of the target. In this way, bowtie‐shaped air‐gaps with ≈4‐nm minimum separation have been attained by Ga FIB milling of a gold film, see Figure 7c,d ^[^ ^72^ ^]^ (Note, these nano‐sized air‐gaps differ from most of the other nanogaps described in this review since they are simply holes in a metal film, and do not divide the metal into two discontinuous parts).

He FIB milling is a more recent technique that offers several advantages over Ga FIB milling for high resolution patterning.^[^ ^61^, ^72^, ^74^, ^75^ ^]^ In contrast to the liquid‐metal ion source used for Ga FIB milling, the He^+^ ions are extracted under high voltage from a sharp, atomically defined metal tip, resulting in a highly directional beam with a typical spot‐size of ≈1.5 nm. The milling rate is typically much lower than for Ga FIB milling due to the lower atomic weight of He, resulting in a slower but more controlled ablation process that can deliver a patterning resolution of 5 nm or better. As a further benefit, He FIB milling typically results in lower levels of contamination since helium is a low‐mass noble gas that can more easily diffuse out of the target material.^[^ ^76^ ^]^ To improve patterning speeds it is sometimes beneficial to use Ga milling and He milling in combination, using Ga ions for initial coarse patterning and He ions for subsequent fine structuring. In this way, Kollmann et al. fabricated bowtie‐shaped gold patterns with nanogap separations as low as 6 nm at the center, see Figure 7e.^[^ ^75^ ^]^

As an alternative to Ga and He milling, it is also possible to use high energy (MeV) beams of protons, neon and krypton to enable higher resolution patterning,^[^ ^62^, ^77^, ^78^ ^]^ although we are not aware of them being used to pattern sub‐10 nm MNGs.

EBL and FIB milling are typically the methods of choice for applications that require the patterning of extremely high‐resolution features over areas of less than a few square microns due to their high resolution, good reliability, and broad versatility in terms of pattern design. However, due to the linear nature of the patterning process, they are too slow for applications that require high resolution patterning over large areas. For large‐area applications, alternative methods such as EUV‐IL (if available) or the emerging techniques discussed in the next section are needed.

## 3 Alternative and Emerging Approaches

In this section, we review several novel approaches to nanogap patterning that differ substantially from the established lithographic methods described above. While these techniques are mostly at an early stage of development and are—with the exception of nanoimprint lithography—still some way from being ready for wide‐scale deployment, they provide simpler routes to nanogap fabrication that are typically faster, more scalable and/or less demanding in terms of the equipment involved than the previously discussed lithographic methods.

### 3.1 Breaking‐ or Cracking‐Based Methods

**Figure 8:**
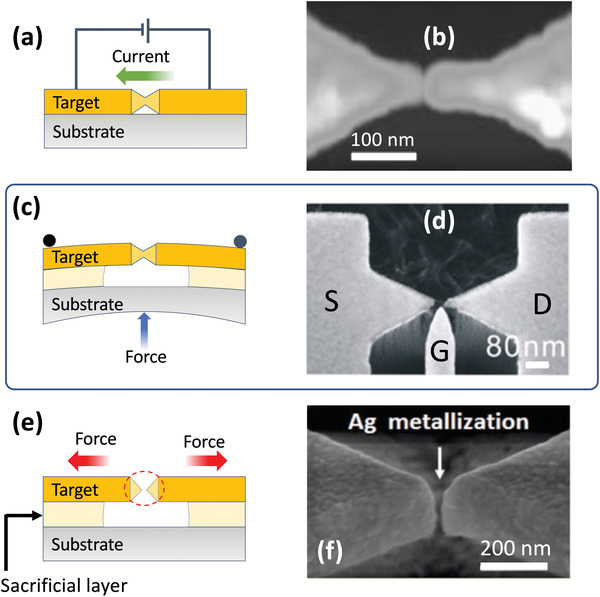
a) Schematic of electromigration‐based break formation, in which a current is passed through a narrow strip of metal that has been lithographically patterned with a central notch. The current induces necking and eventual splitting of the notch due to migration of metal atoms away from the notch region. b) AFM image of gold electrodes obtained by electromigration with a gap width of 1–2 nm. c) Schematic of mechanically controlled breaking, in which a narrow strip of metal with a lithographically patterned central notch is bent on a flexible substrate until the metal splits at the pinch‐point of the notch. By further bending or relaxing the substrate, the width of the gap can be dynamically adjusted. d) Gold source (S) and drain (D) electrodes fabricated by MCB method, with a side‐electrode (G) for gating the current between the other two electrodes. e) Schematic of strain‐induced cracking, in which a rigid substrate is successively coated with a sacrificial layer such as amorphous silicon, followed by a metallized, brittle target material. The target layer is patterned into an array of etched bridges with notches at their mid‐points, and the bridges are then released from the substrate by undercut chemical etching of the sacrificial layer. Removal of the sacrificial layer allows the released target material to contract, and in so doing it splits at the pinch‐point of the notch where the stress is highest. f) SEM image of a sub‐10 nm silver nanogap electrode obtained by stress‐induced cracking. b) Reproduced with permission.^[^ ^86^ ^]^ Copyright 2002, Nature Publishing Group. d) Reproduced with permission.^[^ ^96^ ^]^ Copyright 2013, f) reproduced with permission.^[^ ^106^ ^]^ Copyright 2018, The Royal Society of Chemistry.

In breaking‐ or cracking‐based methods, an initially continuous metal is “split” into two closely spaced electrodes, typically by one of three mechanisms: electromigration (EM) breaking, mechanically controlled breaking (MCB), or strain‐induced cracking. The EM breaking method was introduced by Park et al. in 1999 to study electron transport across gold nanogaps bridged by molecules and colloidal crystals.^[^ ^79^ ^]^ In a typical implementation (see **Figure** **8**a), a thin metal strip of dimensions 15 nm × 100 nm × 300 nm (thickness  × width × length) is first fabricated by conventional lithography. When a high current is passed through the strip, electrical current‐induced diffusion of metal atoms occurs due to momentum transfer from the electrons to the atoms in the metal lattice,^[^ ^3^, ^80^ ^]^ causing the strip to “neck” and eventually “pinch‐off” into two separate parts with a nanoscale clearance. The break formation is sensitive to the manner in which the strip is biased. In one common approach, the strips are subjected to a succession of computer‐controlled linear voltage ramps. Each voltage ramp is ended when the conductance starts to fall, indicating the onset of the EM process. The voltage is then decreased by a small amount (e.g., 100 mV) and a new ramp begins from the new voltage. By carrying out repeated ramps in this way until the conductance reaches a low target value, the EM process can be carried out in an incremental manner that allows gaps as small as a few atoms to be obtained.^[^ ^81^ ^]^

The above EM method suffers from the fact that the applied bias is divided uncontrollably between the strip and the contacts/leads that are used to connect the strip to the voltage source. Significant changes in current density and temperature occur during the EM process, causing considerable instability in the voltage across the strip, which can lead in turn to poor control of the gap‐width.^[^ ^79^, ^82^ ^]^ The reliability of the process can be greatly improved by using a feedback scheme to directly control the voltage across the strip and therefore the power dissipated within it. This may be achieved using a four‐point probe setup to continuously monitor the voltage across the strip while the current flowing through the strip is dynamically adjusted to maintain the voltage at the desired level. Using this and related feedback‐based approaches, it is now possible to routinely control the gap‐width from a few Angstroms to a few nanometers.^[^ ^80^, ^82^, ^83^, ^84^, ^85^, ^86^ ^]^ Park et al. for instance fabricated a transistor with a gap width of 1–2 nm between source and drain (see Figure 8b).^[^ ^86^ ^]^ The EM breaking method has been further extended to the fabrication of small arrays of ultra‐high resolution nanogaps. Johnston et al. for instance fabricated an array of sixteen nanogaps, each having a 1.5 nm gap‐width,^[^ ^82^ ^]^ while Naitoh et al. fabricated an array of ninety nanogaps with sub‐1 nm gap‐widths.^[^ ^87^ ^]^

Mechanically controlled breaking (MCB) is an effective method for fabricating controllable‐width nanogaps between tip‐shaped electrodes.^[^ ^6^, ^88^, ^89^, ^90^, ^91^, ^92^, ^93^, ^94^, ^95^, ^96^, ^97^ ^]^ The technique was originally introduced by Moreland and Ekin in 1985 to study electron tunneling between closely spaced filaments of the brittle, type‐II semiconductor niobium‐tin.^[^ ^98^ ^]^ The method was later extended to non‐brittle materials (such as ordinary metals) by Muller et al.,^[^ ^99^ ^]^ and used to good effect by Reed et al. to study electron transport through a gold/benzene di‐thiol/gold system.^[^ ^100^ ^]^ A schematic of a typical MCB setup is shown in Figure 8c. A strip of metal (typical length of ≈20 mm and width of ≈5 µm) with a lithographically patterned notch at its middle is attached to a flexible substrate. The substrate is fixed at each end to a horizontal rod and—by means of a linear actuator—a vertical force is applied to the middle of the substrate. The substrate flexes and in so doing stretches the strip, causing a progressive reduction in the cross‐section of the notch until it eventually splits into two conically shaped nano‐electrodes. In contrast to nanogaps formed by most other methods, the separation between the electrodes may be dynamically controlled by further bending or relaxing the substrate. Extremely narrow gaps can be achieved in this way. For instance, Perrin et al. reported molecular diodes and transistors based on a gold break junction with variable widths of a few Angstroms or less.^[^ ^97^ ^]^ The diodes were fabricated by forming the break junction directly on a flexible polyimide substrate, while the transistors were fabricated by depositing a layer of oxide‐coated aluminum on the polyimide substrate prior to deposition of the gold electrodes (The aluminum gate electrode was fractured during the cracking step). In both cases, the devices were immersed in a solution of thiol‐terminated zinc‐porphyrin molecules to attach the molecular semiconductor, and the current‐voltage characteristics were used to study charge transport at a single molecule level. In related work, Xiang et al. fabricated gold MCB transistors with tunable gap sizes of around 1 nm, using 1,4‐benzenedithiol as a molecular semiconductor (see Figure 8d).^[^ ^96^ ^]^ In contrast to Perrin et al. they used a separate side‐electrode that remained intact when the break junction was formed.

Although EM breaking and MCB are effective methods for making sharp‐tipped nanogap electrodes down to the sub‐10 nm and even sub‐1 nm level, they can only be used to fabricate small numbers of discrete devices. They are not suitable methods for fabricating high density nanogap arrays over large areas. One breaking‐based technique that can be applied to the fabrication of large‐area arrays is strain‐induced cracking (see Figure 8e).^[^ ^101^, ^102^, ^103^, ^104^, ^105^, ^106^, ^107^ ^]^ In this approach, a rigid substrate is successively coated with a sacrificial layer such as amorphous silicon, followed by a brittle material such as titanium nitride (TiN), and lastly the target metal. The TiN is deposited at high temperature and significant tensile stress builds in the layer as it cools to room temperature. Using a projection stepper system, the TiN/metal layer is patterned into an array of etched bridges with notches at their mid‐points, and the bridges are then released from the substrate by undercut chemical etching of the sacrificial layer, using, for example, isotropic reactive ion etching. Removal of the sacrificial layer allows the released TiN to contract, and in so doing it splits at the pinch‐point of the notch where the stress is highest. This process releases the excess stress and generates a nanogap. Using this approach, Dubois et al. showed it is possible to fabricate large (1 cm^2^) arrays of sub‐3 nm gold nanogaps containing as many as 7 million discrete nanogaps.^[^ ^103^ ^]^

Pan et al. reported a similar approach,^[^ ^106^ ^]^ in which EBL was used to pattern 1‐cm^2^ arrays of tapered silicon nitride (SiN*_x_*) nanobridges on top of a silicon substrate. Parts of the Si substrate were removed by undercut etching with tetramethylammonium hydroxide (TMAH), producing an array of suspended SiN*_x_* nanogaps with gap‐widths of several tens of nanometers. In the final step, the SiN*_x_* nanobridge arrays were metallized with 25 nm of silver by magnetron sputtering deposition, causing the gap‐width to decrease to less than 10 nm (see Figure 8f). Using a tightly focused 532 nm laser as the excitation source (spot‐size 2 µm), individual nanogaps within the array were used as discrete measurement points for Raman detection of Rhodamine 6G dye molecules. Concentrations as low as 10^‐^^16^ M were detectable with large Raman enhancement factors of ≈10^8^.

Strain‐induced cracking represents a substantial step forward in applying cracking‐based methods to large‐area fabrication. In contrast to nanogaps formed by EM breaking and MCB, strain‐based methods have been successfully applied to the fabrication of massively parallel arrays extending over areas up to 1 cm^2^. However, relatively low yields (≈7%) and difficulties in controlling the morphology of the nanogap interfaces remain significant problems. In addition to strain‐induced cracking methods, there are some preliminary reports describing the use of swelling controlled cracking,^[^ ^108^ ^]^ intergranular fracturing,^[^ ^109^ ^]^ and optical breakdown^[^ ^110^ ^]^ which, although relatively unstudied, may have significant potential for array‐based fabrication.

### 3.2 Peeling‐Based Methods

Peeling‐based methods are a group of techniques in which an adhesive material such as a tape or polymer film is applied to the top surface of a metal‐coated substrate, and unwanted parts of the metal are removed in the process of peeling the adhesive from the substrate. Peeling‐based methods typically rely on the unwanted (wanted) parts of the metal film having a stronger (weaker) affinity for the adhesion material than the layer on which they are deposited. In addition to being fast, reliable and scalable to large‐areas, peeling methods also provide a straightforward solution to the challenge of producing nanogaps at the sub‐5 nm level.

Atomic Layer Lithography (ALL) is a nanofabrication technique introduced by Oh and coworkers that uses a combination of Atomic Layer Deposition (ALD) and mechanical peeling to produce ultrathin nanogaps (<5 nm).^[^ ^21^, ^101^, ^111^, ^112^, ^113^, ^114^, ^115^, ^116^, ^117^, ^118^ ^]^ ALD is a self‐limiting form of Chemical Vapor Deposition (CVD) that allows inorganic films of well‐defined thickness to be built up in a step‐by‐step manner. In a typical two‐step ALD reaction, a substrate is placed in an evacuated process chamber. A gas‐phase precursor A is introduced into the chamber and attaches selectively to surface sites on the substrate until the substrate is completely coated. The chamber is then evacuated to remove any unreacted precursor or reaction side‐products, and a second gas‐phase precursor B is introduced into the chamber. B is chosen so that it selectively binds to specific sites on A, allowing B to react with A until all exposed sites are covered and the reaction stops. The chamber is again evacuated to remove unwanted molecules of B, and A is reintroduced to the chamber. If B provides chemically similar sites to those present on the substrate, then the entire process can be repeated (with A first attaching to B until all exposed sites of B are used, and B then attaching to A until all exposed sites of A are used). In this way, the thickness of the deposited layer can be built up in a step‐wise manner, providing atomic level control over the film thickness.

**Figure 9:**
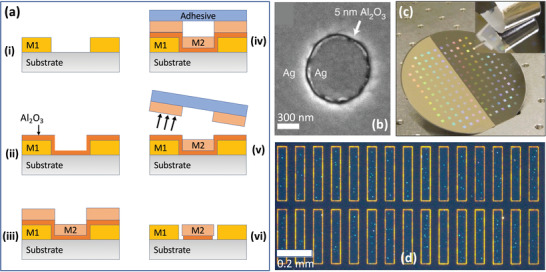
a) Schematic of atomic layer lithography (ALL) process, in which i) a first metal M1 is deposited on a substrate, ii) a conformal layer of Al_2_O_3_ is deposited over the metal and the substrate using atomic layer deposition, iii) a second metal M2 that adheres weakly to Al_2_O_3_ is deposited on top of the coated substrate, iv) a rigid adhesive material is applied to the upper surface of the stack, making contact with the (uppermost) parts of M2 that lie on top of M1, v) the adhesive is peeled away from the stack, taking with it the unwanted parts of M2, and vi) the oxide layer is removed by reactive ion etching, leaving M1 and M2 side‐by‐side on the substrate with a separation equal to the width of the Al_2_O_3_ layer. b) Top‐view SEM image of a 5‐nm‐wide annular gap in a 200‐nm‐thick silver film obtained by ALL. c) Photograph showing a wafer‐scale array of silver nanogap features in a Si wafer. Adhesive tape has been peeled away from the right side of the Si wafer (see inset). d) Optical micrograph of a sample containing approximately 150 000 silver nanogap rectangles (gap size = 5 nm, total ring length = 0.7 mm). b–d) reproduced with permission.^[^ ^116^ ^]^ Copyright 2013, Nature Publishing Group.

In ALL (see **Figure** **9**a), a first metal (“Metal 1,” M1) is patterned on a substrate by conventional lithography (Figure 9a‐i), and a 1–10 nm layer of Al_2_O_3_ is conformally deposited across the full area of the substrate using ALD, coating both the metal and the exposed substrate (Figure 9a‐ii). A second metal (“Metal 2,” M2) of equal height to the first film is then deposited over the full area of the substrate, giving rise to the situation shown schematically in Figure 9a‐iii, where those parts of Metal 2 that lie above Metal 1 are raised with respect to those parts that lie above the substrate. In the cases of noble metals such as gold and silver, the adhesion between the ALD‐deposited oxide layer and the second metal is poor (since it is based on physical adsorption only). Hence, M2 can be removed by applying an adhesive tape across the top‐surface of the substrate (Figure 9a‐iv), and then peeling it away. However, since the adhesive tape used is rather rigid, it can only make good contact with the raised parts of the second metal, and it therefore removes only those parts of Metal 2 that lie above Metal 1 (Figure 9a‐v). Hence, when the peeling process is completed, Metals 1 and 2 are left side‐by‐side on the substrate with a region of Al_2_O_3_ between them whose thickness can be readily controlled by changing the number of ALD cycles. The Al_2_O_3_ in the gap region can subsequently be removed by chemical etching (Figure 9a‐vi) to yield an open nanogap. (The last step may sometimes lead to partial over‐etching of the oxide layer beneath M2 as shown in the diagram).

Figure 9b shows an Ag/Ag metallic nanoring obtained by photolithographically patterning the first metal into a ≈1 µm metal disk before carrying out the rest of the ALL process up to but not including chemical etching of the oxide layer. A 5‐nm layer of Al_2_O_3_ separates the interior (Metal 1) and exterior (Metal 2) silver regions. By using a photolithographically patterned array of 300 µm × 50 µm silver rectangles extending over a 4‐in. glass wafer for Metal 1, Chen et al.^[^ ^116^ ^]^ fabricated an array of 150 000 rectangularly shaped nanogaps of gap‐width 1 nm, demonstrating the inherent scalability of the ALL technique (see Figure 9c,d). The 0.7‐mm perimeter of the rectangular loops rendered them transmissive toward THz radiation, while at the same time the ≈1‐nm gap‐widths resulted in extremely high field enhancements within the gaps due to plasmonic confinement of the field. Hence, the arrays exhibited fifty percent transmission at the resonance wavelength of ≈4 mm despite the gap region covering just 0.002% of the chip area, implying a field‐enhancement factor of ≈25 000.

**Figure 10:**
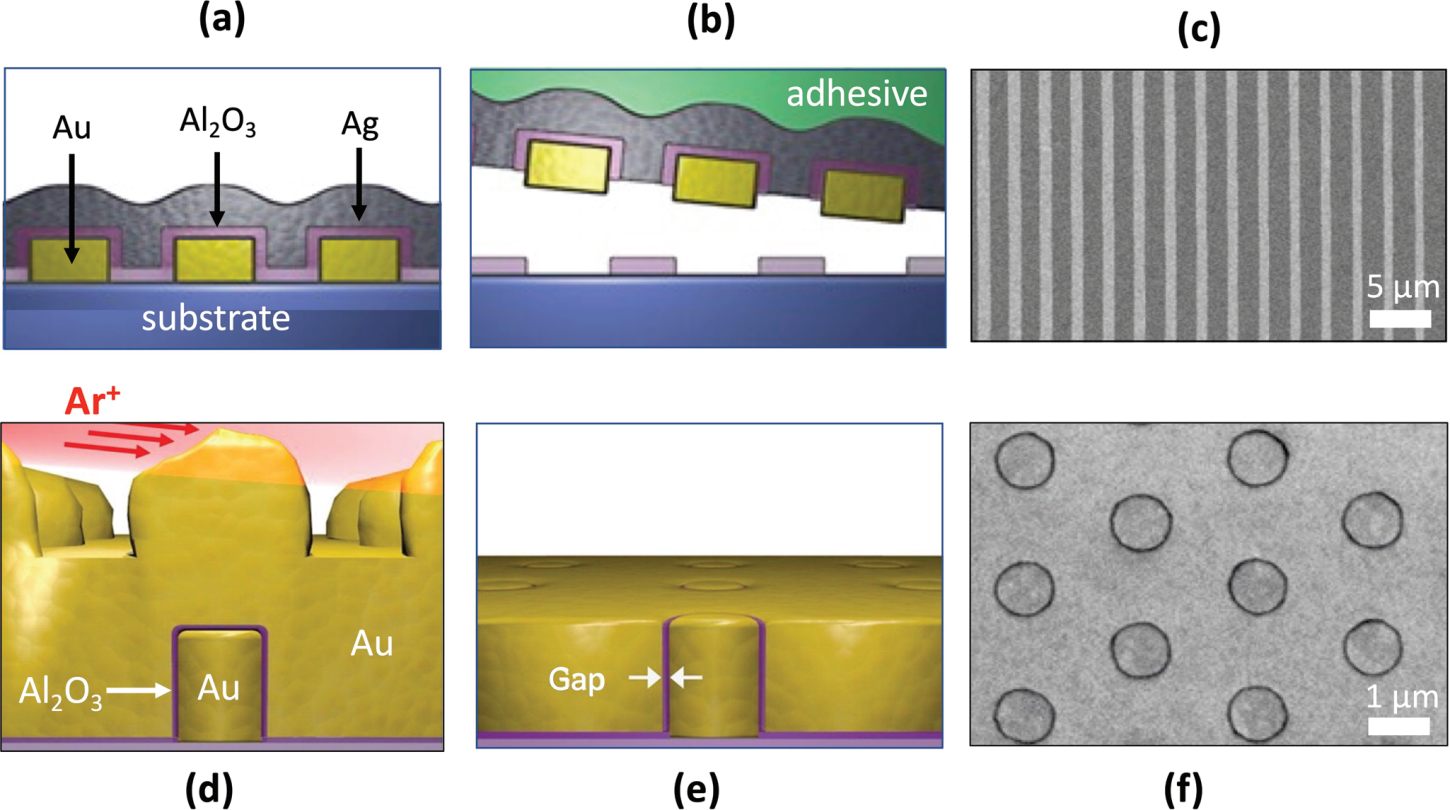
a,b) Procedure for generating smooth metal nanogap arrays by atomic layer lithography. The standard ALL method is followed up to and including the deposition of M2. Then, by choosing an adhesive that conformally coats M2 and binds strongly to it, the complete metal nanogap array is removed from the substrate in the process of peeling away the adhesive, leaving behind only those parts of the Al_2_O_3_ that are directly bound to the substrate. Inverting the adhesive yields a smooth upward‐facing metallic nanogap array, in which the heights of M1 and M2 differ only by the few nanometer thickness of the Al_2_O_3._ c) SEM image of line‐arrays obtained by the modified ALL procedure, with spacings of approximately 5 nm between the Al lines (dark) and the gold lines (light). d,e) Alternative procedure for generating smooth metal nanogap arrays by atomic layer lithography, in which the standard ALL method is followed up to and including the deposition of M2 (d). By subjecting M2 to glancing‐angle ion polishing until the upper surface of M1 is exposed, an ultrasmooth surface is obtained in which the upper surfaces of M1 and M2 are level (e). f) SEM image of resulting nanoring arrays using cylindrically patterned gold for M1 and uniformly deposited gold for M2. The width of the nanorings is approximately 10 nm. a–c) Reproduced with permission.^[^ ^21^ ^]^ Copyright 2015, American Chemical Society. d–f) Reproduced with permission.^[^ ^120^ ^]^ Copyright 2016, American Chemical Society.

One limitation of ALL in its original form is that the upper surface of the nanogap structures is typically uneven due to slight height differences between Metal 1 and Metal 2. For some applications, for example, coupling of near‐field photons into adjacent two‐dimensional materials,^[^ ^119^ ^]^ an ultra‐flat upper surface is required with a surface roughness of a few nanometers or less. Oh and coworkers reported two methods for addressing this need. In the first approach, **Figure** **10**a–c,^[^ ^21^ ^]^ a liquid epoxy was applied to the upper surface of Metal 2, ensuring contact with both its raised and its recessed parts. Once the epoxy had cured, it was peeled from the substrate, taking with it all parts of the metal–dielectric–metal stack, except the Al_2_O_3_ that was chemically bonded to the silicon substrate. Inverting the stripped‐away material yielded a smooth surface with a greatly reduced Metal 1 to Metal 2 height differential equal to the 5‐nm height of the Al_2_O_3_. In the second approach glancing‐incidence ion milling was used to progressively polish away the top metal until the Al_2_O_3_‐filled gaps were exposed, see Figure 10d–f.^[^ ^120^, ^121^ ^]^

Various modifications to standard ALL have been reported, including the replacement of the physical peeling step by chemical etching to improve the yield and the robustness of the final structures,^[^ ^122^ ^]^ the combination of ALL with nanosphere lithography to permit the patterning of large‐area (3 cm^2^) nanogap arrays,^[^ ^123^ ^]^ and the use of optical interference lithography^[^ ^101^, ^124^ ^]^ to pre‐pattern the first metal layer instead of using FIB/EBL methods. Overall, ALL has proven itself to be a versatile and highly scalable method for patterning nanogaps and nanogap arrays with extremely small gap‐widths. Its main limitation is that it is applicable to a relatively narrow palate of weakly adhesive metals such as gold and silver, and in particular it cannot easily be used with metals such as aluminum and titanium that exhibit strong adhesion to typical substrate materials such as silicon and glass.

**Figure 11:**
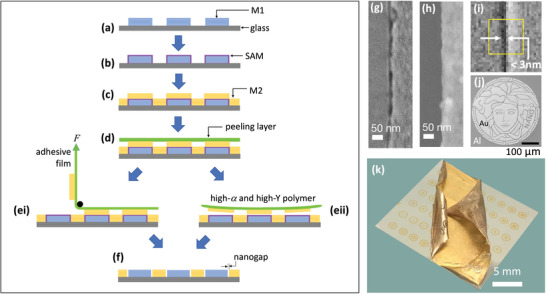
a–f) Schematic showing key processing steps in conventional and self‐peeling adhesion lithography. The conventional procedure comprises the following steps: first, metal M1 is deposited on a substrate and patterned as appropriate (a); second, M1 is selectively coated with a metallophilic SAM (b); third, metal M2 is deposited uniformly over M1 and the exposed substrate (c); fourth, an adhesive film is applied to the surface of M2 (d); fifth, the film is peeled away from the substrate, selectively removing M2 from those regions located directly above the SAM (e‐i); finally, the SAM is removed by UV/ozone or O_2_‐plasma treatment, leaving M1 and M2 sitting in a complementary arrangement side‐by‐side on the substrate (f), separated in the limiting case by the length of the SAM. The self‐peeling procedure follows the conventional method up to step (d), except the peeling layer comprises a polymer with a high coefficient of thermal expansion *α* and a high Young's modulus *Y*, spin‐coated onto M2 from a heated solution. As the polymer film cools, tension builds inside the film until it is sufficient to induce spontaneous peeling of the polymer from the coated substrate, taking with it those parts of M2 that are located directly above the SAM (e‐ii). The SAM is removed as before by UV/ozone or O_2_‐plasma treatment. SEM images of Au‐Al nanogaps obtained with g) matched and h) unmatched metal heights, using a single layer of ODT as the SAM, 50‐nm gold for M1 and 50‐nm Al or 30‐nm Al for M2. i) High resolution SEM image of a sub‐3 nm Al/Au nanogap. j) SEM image showing an example of large‐area patterning using adhesion lithography. The separation between the light regions (Au) and dark regions (Al) is approximately 5 nm. k) Photograph showing formation of Al/Au nanogap arrays by self‐peeling adhesion lithography. The photograph was taken midway through the peeling step, with the peeling layer partially detached from the metal‐coated substrate. (a‐f,k) Adapted under the terms of the Creative Commons CC‐BY license.^[^ ^130^ ^]^ Copyright 2019, The Authors. Published by Wiley‐VCH. g‐j) Adapted under the terms of the Creative Commons CC‐BY license.^[^ ^131^ ^]^ Copyright 2021, The Authors. Published by Wiley‐VCH.

Adhesion lithography (“a‐lith”) is a nanofabrication technique introduced by Beesley et al. that, like ALL, uses peeling to remove unwanted parts of a deposited metal.^[^ ^125^, ^126^, ^127^, ^128^, ^129^ ^]^ As illustrated in **Figure** **11**a–f, a prepatterned metal film (M1) is deposited onto a substrate by photolithography or by shadow mask evaporation (Figure 11a). An alkyl‐terminated self‐assembled monolayer (SAM) is then conformally coated onto the exposed surfaces of M1 (Figure 11b) by immersing the substrate in a solution of the SAM molecules. The head group of the SAM is selected so that it attaches only to the surface of M1 and not to the substrate, which is assumed here to be glass or silicon. A thiol head group is commonly used when attaching to gold, while a phosphonic acid head group is suitable when attaching to oxide‐coated metals such as aluminum or titanium. After cleaning the substrate to remove all unbound SAM molecules, a second metal film (M2) is uniformly deposited over the entire (or selected parts of) the substrate (Figure 11c). Owing to the presence of the SAM, the adhesion of M2 to M1 is much weaker than its adhesion to the substrate. In consequence, if an adhesive material is applied uniformly to the surface of M2 (Figure 11d) and then peeled away, M2 will detach from the regions above M1, leaving the first and second metals side‐by‐side on the substrate, with a self‐assembled monolayer between them (Figure 11e‐i). Treatment with oxygen plasma or UV/ozone removes the self‐assembled monolayer, resulting in an open metallic nanogap (Figure 11f). At the end of the procedure, the two metals M1 and M2 sit in a complementary arrangement side‐by‐side on the substrate, separated in the limiting case by the length of the SAM, that is, a few nanometers or less.

Using a‐lith in the form in which it was originally developed, the obtained gap‐widths are typically in the range of 10–30 nm, substantially larger than the length of the SAM molecule (see, e.g., Figure 11g). The key to achieving narrow gap widths via a‐lith is to ensure clean splitting of the second metal along the lines where the wanted and unwanted parts of M2 must be separated, that is, along the edge profile of M1. Any tearing of M2 during peeling risks widening the gap‐width undesirably. To achieve a clean split, the second metal must be pre‐fractured before peeling takes place, and any forces applied during the peeling step should be minimized. Luo et al. showed that pre‐fracturing may be achieved by making the second metal substantially thinner than the first metal (e.g., 30 nm vs 50 nm), which prevents M2 from conformally coating the terrain of the M1‐patterned substrate, automatically leading to breaks in M2 along the edge profile of M1.^[^ ^131^ ^]^ In this way, they were able to reduce the gap width to less than 3 nm—only slightly higher than the 2‐nm length of the SAM molecule employed (Figure 11h,i). Figure 11j shows an SEM image of “Medusa” fabricated in gold and aluminum by a‐lith using a single layer of 1‐octadecanethiol (ODT) as a spacer/SAM. The image extends over several hundred microns in each direction and sub‐5 nm gaps exists at the Au/Al interfaces, demonstrating the ability of a‐lith to pattern nanogaps over large areas. In related work^[^ ^130^ ^]^ Luo et al. showed that inadvertent widening of the gap during the peeling step could be avoided by using (for the peeling layer) a thermoplastic fluoropolymer such as polyvinylidene fluoride (PVDF), which has both a high coefficient of thermal expansion *α* and a high Young's modulus *Y*. When a film of PVDF is spin‐cast onto M2 from a heated solution and allowed to cool, significant stress builds inside the layer until it spontaneously delaminates from the underlying (coated) substrate, taking with it the unwanted parts of M2 that lie directly above M1 (see Figure 11e‐ii,k). Importantly, it can be shown that no vertical forces are exerted on the substrate by the peeling layer during the delamination process (and the only horizontal forces are tensile forces that pull M1 and M2 together).^[^ ^130^ ^]^ Consequently, the use of PVDF as a peeling layer is a simple way to prevent accidental widening of the nanogap during the peeling process.

Luo et al. further showed that, by varying the length of the spacer molecule, it is possible to tune the electrode spacing from <3 nm to >30 nm.^[^ ^131^ ^]^ They replaced the SAMs used in standard a‐lith by extendable chains of metal‐ligated self‐assembled multilayers, known as molecular rulers.^[^ ^132^, ^133^, ^134^ ^]^ The molecular rulers were formed using SAM molecules with thiol head groups and carboxylic acid end groups by alternately immersing a gold‐coated substrate in ethanolic solutions of the SAM molecules and copper perchlorate. In the first step—using gold for M1—the thiol SAM molecules were conformally attached to the patterned gold, with the carboxylic acid groups facing outward. In the second step, Cu(II) ions were coordinated with the carboxylic acid groups of the first self‐assembled monolayer, forming an atomically thin layer that served as a linker upon which a second thiol SAM could be conformally attached. With each cycle, an additional SAM was added to the multilayer, increasing the layer thickness—and hence the final gap size—by approximately 2 nm.

**Figure 12:**
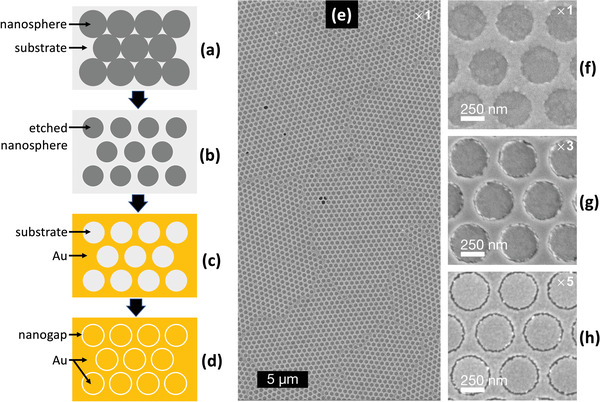
Fabrication of massively parallel nanoring arrays using a combination of NSL and size‐tunable a‐lith.^[^ ^131^ ^]^ a–d) Schematic of the fabrication procedure, in which: first, a monolayer of close‐packed polystyrene nanospheres is deposited on a substrate (a); second, the nanospheres are “shrunk” by oxygen plasma treatment, leaving voids between them (b); third, metal M1 = Au is deposited on the substrate through the nanosphere template and the template is removed, leaving a hexagonal array of nanoholes in the gold film (c); and, fourth, the holes are “filled” with a second metal (M2 = Au) using size‐tuneable adhesion lithography, resulting in a hexagonal array of ring‐shaped nanogaps (d). e) 20‐µm × 40‐µm SEM image of an Au–Au nanoring array, obtained using a molecular ruler of length *N* = 1. f–h) High magnification SEM images of Au‐Au nanoring arrays, obtained using molecular rulers of length *N* = 1 (f), *N* = 2 (g), and *N* = 5 (h). Each array has a pitch of ≈500 nm and a ring‐diameter of ≈380 nm, defined by the nanosphere diameters before and after etching. Reproduced under the terms of the Creative Commons CC‐BY license.^131^ Copyright 2021, The Authors. Published by Wiley‐VCH.

One favorable feature of peeling‐based methods is their scalability to large areas, which opens up the possibility of fabricating massively parallel arrays of nanogaps. As a proof of concept, Luo et al. fabricated large‐area (≈50 mm^2^) arrays of nanorings by combining a‐lith with a soft colloidal lithography method known as nanosphere lithography (NSL, see **Figure** **12**a–d).^131^ In brief, the combined technique comprised the following steps: a hexagonal close‐packed monolayer of polystyrene (PS) spheres was deposited on a substrate by drop‐casting (Figure 12a); the nanospheres were then isotropically “shrunk” via oxygen plasma etching so they no longer touched (Figure 12b); the first metal was evaporated onto the substrate through the spaces between the spheres; and the spheres were then removed, leaving the first metal patterned with a hexagonal array of circular holes (Figure 12c); continuing the size‐tunable a‐lith method from this point filled in the holes with a second metal and yielded a macroscopic array of near‐identical ring‐shaped nanogaps (RSNs, Figure 12d), in which the pitch, diameter and gap‐width of the RSNs were respectively determined by the initial diameter of the PS spheres, the etched diameter of the PS spheres, and the number of layers used in the molecular ruler.

For a typical drop‐cast area of 50 mm^2^ and an initial nanosphere diameter of around 500 nm, each array contained some 200 million discrete and nominally identical nanorings—thought to be the largest nanogap arrays reported to date (in terms of the total number of discrete nanogaps). Like any method based on nanosphere lithography, defects such as dislocations and vacancies in the initial nanosphere template introduce some disorder into the final pattern. However, executed with care, the combined NSL/a‐lith method provides a simple means of rapidly fabricating well‐ordered and massively parallel arrays of nearly identical nanogaps that extend over multi‐millimeter length‐scales with relatively low defect densities, see, for example, Figure 12e. Nanorings obtained using equally sized PS spheres with molecular rulers of length 1, 3, and 5 are shown in Figure 12f–h, with a progressive increase in gap size from <3 nm to ≈10 nm as the molecular ruler length increases.

Key advantages of the a‐lith technique include: the ability to fabricate co‐planar nanogaps between dissimilar conductive materials with extremely large (length‐to‐width) aspect ratios in excess of 10^6^; compatibility with a broad range of substrate materials like glass, silicon, and plastic; and easy integration with many other nanofabrication methods, such as photolithography and nanosphere lithography. These advantages have been exploited in a variety of nanogap devices, including Schottky junctions/diodes,^[^ ^126^, ^129^, ^135^ ^]^ photodetectors,^[^ ^136^, ^137^ ^]^ OLEDs,^[^ ^127^, ^136^, ^138^ ^]^ transistors,^[^ ^128^, ^138^ ^]^ memristors,^[^ ^138^, ^139^, ^140^ ^]^ and bio/chemical sensors.^[^ ^131^ ^]^ A current limitation of the a‐lith method is the difficulty of reliably accessing ultra‐narrow gap widths below 1 nm.

The a‐lith process bears some similarity to ALL, but there are several notable differences. In the case of ALL, the entirety of the second metal is deposited onto the same material, namely an oxide layer, meaning there is no engineered spatial variation in the strength of adhesion to the underlying layer. To successfully pattern M2 by ALL, the adhesive should therefore come into contact only with the unwanted parts of M2 (i.e., those parts that sit directly above M1), which requires a rather inflexible adhesive film that will not conformally coat the surface of M2. In the case of a‐lith, by contrast, conformal coating of M2 is the preferred choice for reliable patterning, and the selective peeling of M2 occurs due to a difference in the strengths of adhesion between M2 and the SAM and M2 and the substrate. Following patterning by a‐lith, M2 is in direct contact with the substrate, whereas in the case of ALL a thin oxide layer separates M2 from the substrate. From a practical perspective, ALL requires access to an ALD coater and is limited to weakly adhesive noble metals such as gold and silver; a‐lith uses simple solution processing for deposition of the SAM spacer, and is applicable to a wide variety of metals. On the other hand, ALL has been used to achieve sub‐1 nm nanogaps, whereas the ultimate resolution of a‐lith is determined by the >1 nm width of the spacer molecules.

**Figure 13:**
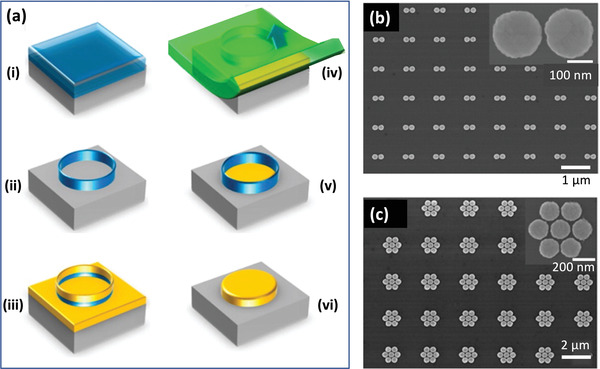
a) Schematic of SPLprocess,^[^ ^141^ ^]^ in which i) a layer of HSQ negative e‐beam resist is deposited on a substrate, ii) a circular pattern is defined in the resist by EBL, iii) a thin gold layer is evaporated onto the resist‐coated substrate, iv) an adhesive polymer is drop‐cast onto the substrate and cured under UV irradiation, v) the adhesive is peeled from the substrate, leaving gold trapped inside the ring of resist, and vi) the resist is removed by a developer, leaving a disk of gold. b) SEM images of plasmonic gold structures obtained by using EBL to define “figures of eight” (i.e., pairs of touching gold rings) in the resist at step (a‐ii). c) SEM images of plasmonic gold structures obtained by using EBL to define clusters of seven touching rings in the resist at step (a‐ii). The gaps between the gold discs are approximately 15 nm. Reproduced with permission.^[^ ^141^ ^]^ Copyright 2016, American Chemical Society.

Sketch and peel lithography (SPL) is a peeling‐based method introduced by Duan and co‐workers that may be applied to metals that adhere weakly to a substrate, for example, gold on glass.^[^ ^141^, ^142^ ^]^ In the first stage of the SPL process, a layer of resist is deposited on a substrate (**Figure** **13**a‐i), and a closed outline of a target feature is then “written” into a negative resist via EBL. If the target feature is a disc, for instance, a ring is drawn around the circumference of the disc. The sample is then immersed in a developer, which causes the unexposed resist to be removed, leaving a ring of insoluble resist (Figure 13a‐ii). A metal film of lower thickness than the resist is deposited over the full area of the substrate causing the metal to divide into three parts located inside, outside and on top of the ring (Figure 13a‐iii). Next, an adhesive film is applied on top of the metal and then peeled away (Figure 13a‐iv), leaving metal only in the regions enclosed by the ring‐shaped resist where the gold is difficult to remove and becomes “trapped” (Figure 13a‐v) (Note, the reasons for the metal remaining inside the ring are unclear. Pointing to theoretical studies by Sarkar et al.^[^ ^163^ ^]^ and Shull et al.^[^ ^164^ ^]^ Chen et al. argue that the adhesive is likely to exist in a state of high stress inside the ring, which would greatly reduce the force *f*_1_ needed to detach it from the gold film. They argue that, inside the ring, *f*_1_ is smaller than the force *f*_2_ needed to detach gold from the substrate, and hence delamination occurs at the adhesive/gold interface. Outside and on top of the ring, the stresses in the adhesive are thought to be much lower, resulting in a much higher value for *f*_1_ that exceeds *f*_2_ and therefore causes delamination at the substrate/gold interface.^[^ ^141^, ^142^ ^]^).Finally, the resist is removed by immersion in a developer (Figure 13a‐vi). If a number of touching rings are drawn in the photoresist during the exposure step, then closely spaced discs are obtained after the peeling step, with a nanogap between them (see, e.g., Figure 13b,c). Importantly, since it is only necessary to expose the outlines of the target features (which typically account for just a small fraction of the total substrate area), the method is relatively quick, and arrays of a few thousand features can be readily fabricated. The main requirements for reliable patterning during the peeling step are that all outlines should be closed (with no physical breaks in the photoresist) and the areas enclosed by the outlines should extend no more than a few tens of microns in any direction. This latter requirement precludes certain large‐area applications such as the THz resonators discussed above, but it has been shown that by using ultrasonication instead of peeling to remove the unwanted metal, multiscale metallic patterns of up to 1‐mm may be obtained with sub‐10 nm gaps between individual features.^[^ ^143^ ^]^

**Figure 14:**
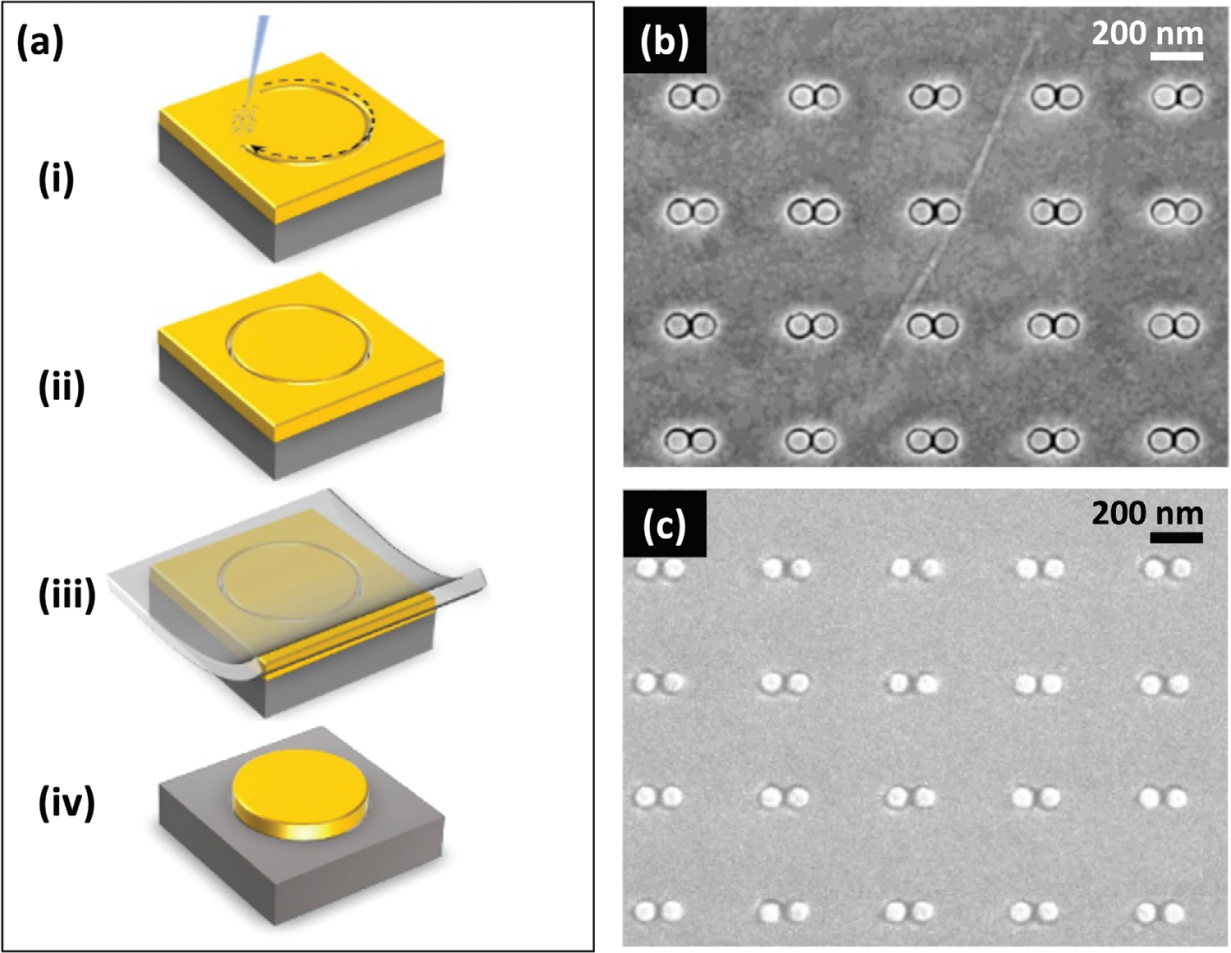
a) Schematic showing SPL by FIB milling,^[^ ^142^ ^]^ in which i,ii) a narrow ring‐shaped trench is milled directly into a metal film, iii) a layer of adhesive is applied to the metal film, and iv) the adhesive is peeled from the metal film, taking with it all metal outside of the trench. b,c) SEM images of gold dimers formed by using helium FIB‐milling to define “figures of eight” (i.e., pairs of touching gold rings) in a gold film at step (i). The images show the situation b) before and c) after peeling. The separation of the discs is approximately 15 nm. Reproduced with permission.^[^ ^142^ ^]^ Copyright 2016, American Chemical Society.

A number of variations to the SPL procedure have been reported.^[^ ^144^, ^145^, ^146^, ^147^, ^148^ ^]^ For instance, instead of using a negative resist, it is possible to directly create nano‐trenches in a deposited metal film by FIB milling before selectively removing the metal outside of the trenches by peeling, see **Figure** **14**.^[^ ^142^ ^]^ In the case of a silicon substrate, it has been shown that slight over‐milling of the unwanted metal causes a thin layer of silicon atoms to be sputtered onto the side walls of the retained metal, which serves to protect the retained metal from damage during the subsequent peeling step. SPL has also been extended to strongly adhesive metals such as aluminum by depositing a fluorine‐based self‐assembled monolayer on top of the substrate to modify its surface energy.^[^ ^145^ ^]^ All in all, SPL is a versatile and reliable method for nanogap patterning that provides a good balance between speed and throughput as it requires only the outline of the patterns to be defined in the photoresist. However, it is best suited to weakly adhesive metals such as gold and silver or substrates treated with adhesion‐reducing surface modifiers, which may potentially compromise the mechanical stability of the resultant nanostructures.^[^ ^149^ ^]^

### 3.3 Nanoimprinting Methods

**Figure 15:**
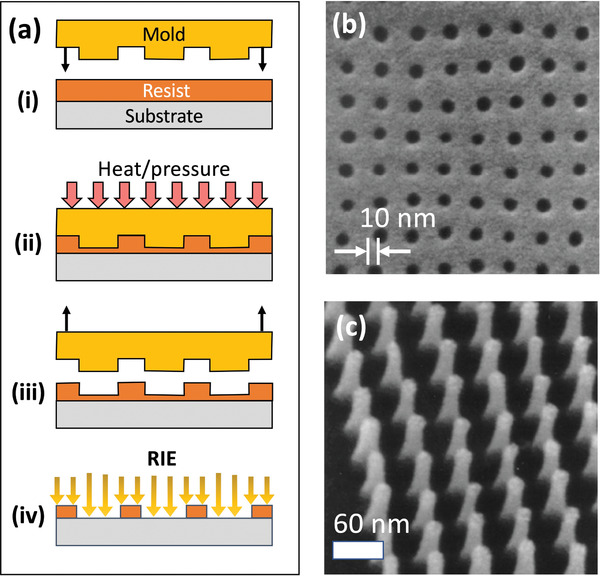
a) Schematic of thermal nanoimprint lithography (NIL) process, in which i,ii) a rigid mold is pressed into a resist to create a thickness contrast, iii) the mold is mechanically separated from the thermally‐cured resist, and iv) the compressed resist is anisotropically etched away, leaving resist only in the locations where imprinting did not take place. b) Hole array imprinted in poly(methyl methacrylate) (PMMA) with ≈10 nm diameter holes.^[^ ^151^ ^]^ c) SEM image of Si mold used to generate the pattern shown in (b), comprising a regular 2D‐array of columns of approximate width 10 nm. b,c) Reproduced with permission.^[^ ^151^ ^]^ Copyright 1997, American Institute of Physics.

Thermal nanoimprint lithography (NIL) is a physical patterning technique that uses mechanical embossing to create a relief pattern in a resist.^[^ ^150^, ^151^, ^152^, ^153^, ^154^ ^]^ In a typical implementation, a nanostructured rigid mold is pressed into a thin layer of resist on a substrate to create a thickness contrast (**Figure** **15**a‐i), and the resist is hardened by thermal annealing (Figure 15a‐ii). The mold is mechanically separated from the thermally cured resist (Figure 15a‐iii), stepped to a new position for another imprint and curing step, and the process is repeated until the full area has been patterned. In the last step, the resist is anisotropically thinned by reactive ion etching until the underlying substrate is exposed, leaving resist in the unimprinted locations only (Figure 15a‐iv). The imprinted resist may then be used for pattern transfer to a metal in the usual way, for example, by shadow mask deposition. NIL provides a convenient and rapid method for replicating nanoscale features over large areas. Figure 15b for instance shows a large‐area array of 10‐nm nanoholes formed in a 78‐nm layer of the thermoplastic poly(methylmethacrylate) (PMMA), using a rigid silicon mold structured with an array of 10‐nm‐diameter pillars (Figure 15c).^[^ ^151^ ^]^

**Figure 16:**
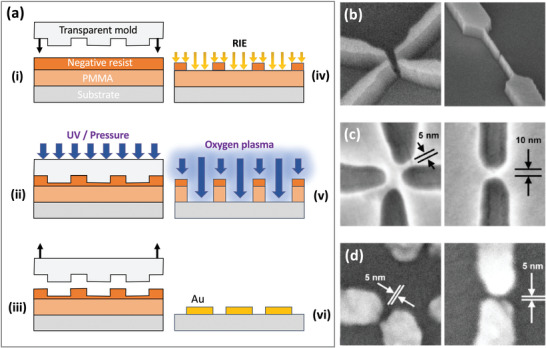
a) Schematic of photocurable nanoimprint lithography, in which i) a transparent silica mold is pressed into a bilayer of poly(methyl methacrylate) (PMMA, transfer polymer) and a negative resist, ii) the resist is exposed to UV light, causing it to harden, iii) the mold is removed, iv) the resist is subjected to reactive ion etching until the PMMA layer is exposed, v) the exposed PMMA is etched away by treatment with an oxygen plasma, and vi) the patterned bilayer of PMMA and resist is used as a shadow mask for metal deposition. The bilayer may alternatively be used as an etch mask. b) SEM images of silicon oxide molds used for photocurable NIL. c) SEM images of contact shadow masks, obtained using the molds in (b). d) SEM images of gold nanogaps, obtained using the contact masks in (c). b–d) Reproduced with permission.^[^ ^150^ ^]^ Copyright 2004, American Institute of Physics.

In a simple variation of NIL a UV‐curable negative resist is used to define the pattern, and the exposure step is carried out simultaneously with the embossing step by illuminating the resist through a transparent mold.^[^ ^153^, ^154^ ^]^ Using this approach Austin et al. fabricated gold nanogaps with gap‐widths as low as 5 nm by depositing an interlayer of PMMA between the substrate and the UV‐curable resist, see **Figure** **16**a.^[^ ^150^ ^]^ The resist was imprinted and exposed using a silicon oxide mold (steps i to iii of Figure 16a), and then subjected to reactive ion etching until the PMMA layer was exposed (Figure 16a‐iv). The coated substrate was then treated with an oxygen plasma (Figure 16a‐v), which had the effect of etching the exposed PMMA all the way down to the substrate surface, while leaving the (more resilient) resist largely intact. Hence trenches were formed in the bilayer polymer at the locations where the resist was originally imprinted. Using the patterned bilayer film as a contact mask, a thin layer of metal (Cr/Au) was deposited over the full area of the substrate. Finally, the substrate was washed with warm acetone, causing the PMMA to dissolve and the resist/metal bilayer to detach from the substrate. At the end of the procedure, a small amount of Cr/Au was left behind on the substrate at the locations where the resist was originally imprinted (Figure 16a‐vi). In this way nanogaps with spacings as low as 5 nm were obtained, matching the feature size in the NIL mold (see Figure 16b–d). Austin et al. further demonstrated the scalability of the approach by patterning high quality 200‐nm gratings over a four‐inch silicon wafer.

NIL has many advantages compared to techniques such as photolithography and EBL. The resolution of NIL is unaffected by factors such as wave diffraction, optical scattering due to inhomogeneities in the resist, back‐scattering from the substrate and chemical effects that affect the patterning resolution. The principal drawback is that the NIL mold must be patterned by conventional means such as EBL, which can be slow and costly depending on the required size. Hence, it is best suited to applications where the nanogap design is fixed, and many replicates of exactly the same geometry are required. In such circumstances, mold fabrication is a one‐off inconvenience, and multiple daughter molds may be prepared from the original master mold. With a preprepared mold in hand, NIL is a fast, scalable and reliable technique that allows for patterning over large areas with minimal defects.

## 4 Summary and Outlook

In summary, a wide range of methods have been developed to fabricate nanogap electrodes that variously use optical, electromagnetic, mechanical, and chemical principles to carry out the patterning step. The state‐of‐the‐art in large‐area patterning at the sub‐10 nm level is EUV‐PL —a technique that combines high resolution with high throughput. However, the extremely high cost of the technology means it is unlikely to expand beyond commercial (high‐end) chip manufacturing for many years to come. EUV‐IL based on the interference of coherent diffracted EUV light‐beams is a more viable technique for research purposes that also offers high resolution and high throughput, although it is limited to 1D line arrays, 2D spot arrays and other relatively simple periodic structures, and hence it cannot provide the breadth of feature shapes offered by EUV‐PL. The need for EUV light in the photolithographic patterning of nanogaps can often be avoided by using techniques that side‐step the Abbe diffraction limit, exploiting the fact that the feature size in a patterned resist may be smaller than the spot‐size of the illuminating radiation. The best established of these side‐stepping techniques are multibeam interference lithography and multiple patterning methods. UV/Vis multibeam interference lithography is a fast, reliable, and highly scalable patterning method, but it is limited in terms of the achievable feature shapes and to‐date has not been applied to sub‐10 nm nanogap arrays. Multiple patterning is a well‐established method for fabricating nanoscale features that—by splitting a dense pattern across several sparser masks—gets around the Abbe limit at the expense of additional lithographic steps and the need for extremely good registration between steps. UV/Vis DLW using thermal resists is an intriguing yet underexplored technique for patterning arbitrarily‐shaped nanoscale features that (like other DLW lithographies) has the major advantage of requiring no pre‐fabricated mask and therefore permitting rapid prototyping. Importantly, the technique could potentially bring nanogap fabrication facilities to any cleanroom with existing DLW equipment, the key change relative to standard DLW being the use of a non‐linear thermal resist.

The principle advantages of optical techniques are that they are fast, reliable, and scalable to large areas, while their main disadvantage is an exponential increase in the complexity (and cost) of the patterning procedure as the feature sizes approach the sub‐10 nm level.

In contrast to optical methods, charged particle‐based methods provide easy access to sub‐10 nm features, using equipment that can be routinely found in nanofabrication facilities across the world. EBL and FIB milling represent the state‐of‐the‐art for nanoscale patterning at a research level, and are capable of providing exceptionally high quality patterning down to the few nanometer level. The linear nature of the patterning procedure means they are best suited to small‐area applications below a few tens of square microns, although they also play a critical supporting role in many large‐area patterning methods. For example, the physical structuring of break junctions prior to the breaking step is frequently carried out by EBL or FIB milling while, in NIL, it is usual to fabricate the silicon master mold by EBL.

NIL is probably the most appropriate alternative technique for large‐area patterning of nanogaps where the design pattern is fixed and there is a need for fast, high fidelity replication of that pattern. Break junctions are likely of most value as research tools due to their ability to access extremely low (sub‐nanometer) gap‐widths and the ability in some cases to dynamically control the gap‐width. Peeling‐based methods offer perhaps the most attractive approach for rapidly prototyping sub‐10 nm nanogaps over large areas (>1 mm^2^). The two most widely studied peeling methods—ALL and a‐lith—have the advantage of not requiring EBL or FIB patterning at any point in the fabrication process: the first metal may be patterned by a wide variety of lithographic processes, while the second (possibly different) metal is automatically deposited in the voids between the first metal without any lithographic patterning step. Both methods offer similar advantages in terms of speed and scalability, although ALL is currently able to access narrower nanogaps than a‐lith, while a‐lith has less demanding equipment requirements and is applicable to a broader range of metals. For moderate‐area applications, SPL provides a convenient, reliable and fairly rapid fabrication method whose throughput is largely determined by the FIB or EB system used to draw‐out the nanogap profiles. In contrast to ALL and a‐lith, SPL is restricted to symmetric nanogaps, in which both electrodes are formed from the same metal; consequently it cannot be applied to the fabrication of asymmetric nanoscale devices such as rectifiers and ambipolar devices that require the use of closely spaced dissimilar metal electrodes.

There are many other approaches to nanogap patterning that we have not addressed in this article (although we have tried to cover most methods that have been extensively applied to large‐area patterning). In **Table** **1** we provide an overview of the techniques reviewed above, while in **Table** **2** we provide similar information for other (less scalable) patterning techniques. We caution that no single method should be viewed as a contender for the title of “best nanogap patterning method.” Each one offers its own advantages and disadvantages—in terms of speed, reliability, scalability, cost, materials compatibility, ease of implementation, and versatility—and the most appropriate choice of technique depends entirely on the application at hand. Moreover, many of the most interesting opportunities for nanogap patterning come from the combination of methods, for example, the combination of peeling‐based methods with nanosphere lithography, interference lithography, or nanoimprint lithography.

**Table 1:** Summary of nanogap fabrication methods discussed in this review article

Technique	Minimum gap size [nm]	Throughput (Low, Medium, High)	Parallel/Serial	Cost	Refs
EUV photolithography	≈10	H	P	H	^[^ ^40^ ^]^
UV/vis multibeam interference lithography	>10	H	P	L	^[^ ^47^ ^]^
Multiple patterning	~10	H	P (at each step)	M	^50^
Direct laser writing	≈5	M/H	S	M	^[^ ^45^ ^]^
E‐beam lithography	≈5	M	S	H	^[^ ^60^ ^]^
E‐beam milling	<1	L	S	H	^[^ ^67^ ^]^
FIB milling	≈3	M/L	S	H	^[^ ^109^ ^]^
Electromigration	≈1	L	S	M	^[^ ^86^ ^]^
Mechanical breaking	≈1	L	S	H	^[^ ^96^ ^]^
Crack junction	<3	M	P	M	^[^ ^103^ ^]^
Atomic layer lithography	<1	H	P	M	^[^ ^116^ ^]^
Adhesion lithography	<3	H	P	L	^[^ ^131^ ^]^
“Sketch and peel” lithography	≈5	M	Partly parallel	H	^[^ ^146^ ^]^
Nanoimprint lithography	≈5	H	P	M	^[^ ^150^ ^]^

**Table 2:** Overview of nanogap fabrication methods not discussed in this review article

Technique	Minimum gap size [nm]	Throughput (Low, Medium, High)	Parallel/Serial	Cost	Overview	Refs
Plasmonic lithography	≈10	H	P	H	A metal mask containing nanoscale features is placed in contact with a photoresist. Light incident on the mask is “squeezed” into dimensions far below the diffraction limit due to coupling with surface plasmons (oscillating surface electrons at the metal/resist interface). The resulting electric field hot spots lead to highly localized exposure of the resist, generating nanoscale features. Requires a pre‐patterned nanostructured mask.	^[^ ^54^ ^]^
Nanoparticle self‐assembly	<1	L	P	L	A dilute dispersion of gold colloids is deposited on a substrate. Individual gold dimers with different gap sizes are randomly formed as the solvent evaporates. Fixed feature shape, non‐scalable, gap‐width, and positioning on the substrate are not controlled.	^[^ ^155^ ^]^
Block copolymer lithography	5	M	P	L	A hexagonal array of holes in a thin metal film is fabricated by nanosphere lithography. Si‐containing block copolymers (BCPs) self‐assemble inside the holes in a concentric manner. Plasma oxidation of the assembled BCPs leads to the formation of concentric SiO*_x_* nanorings. A thin layer of gold is deposited onto the substrate and the SiO*_x_* is removed by reactive ion etching, leaving a concentric series of gold rings inside each hole with a ring‐to‐ring separation of a few nanometers. Feature shape (rings) is fixed.	^[^ ^156^ ^]^
Nanosphere lithography	≈5	M	P	L	A monolayer of hexagonally packed nanospheres is deposited on a substrate and lightly etched using oxygen plasma to create a nanogap separation between the spheres. The spheres are used as an evaporative contact mask for metal deposition, resulting in nanogaps in the locations where the spheres previously touched. Feature shape fixed.	^[^ ^157^ ^]^
Scanning probe lithography	3	M	S	M	A sharp scanning probe induces local conversion of a resist via physical or chemical means, enabling subsequent nanoscale patterning of a metal.	^[^ ^158^ ^]^
Dip pen lithography	≈12	L	S	M	A coated AFM tip delivers protective molecules to a metal surface. The tip is momentarily raised as it is linearly scanned across the surface, inducing a nanoscale break in the line of deposited molecules. Chemical etching removes all exposed metal, leaving a broken metal line underneath the deposited molecules with a nanoscale gap between the two parts.	^[^ ^159^ ^]^
On‐wire lithography	<5	M	P	M	A‐B‐A segmented nanowires are formed electrochemically from two metals A and B, where the width *w* of the B segment determines the final gap width. The wires are transferred to a substrate and capped with a protective layer of silica. The coated wires are detached from the substrate by ultrasonication and subjected to a wet‐chemical etching procedure that preferentially removes B, leaving a nanogap of width *w* between the two A segments. The silica coating holds the two segments of A in place. Limited to the formation of nanogaps in nanowires.	^[^ ^160^ ^]^
DNA origami	<5	L	S	H	DNA self‐assembly methods are used to create bow‐tie‐shaped DNA templates with a nanogap at their centers. The templates are mixed in solution with thiolated‐DNA‐capped gold nanoparticles, causing the nanoparticles to attach to the DNA templates. The resulting gold nanostructures mirror the bow‐tie shape of the original templates, with a nanogap remaining at the center. The gold‐coated templates are transferred to a substrate by, for example, drop‐casting. Placement on the substrate is not controlled.	^[^ ^161^ ^]^
Nanoskiving	<1	L	S	H	A metal/graphene/metal (m/g/m) sandwich structure is formed on a substrate, released from the substrate, and then embedded in a block of epoxy. An ultramicrotome removes a thin slice of the epoxy block, cutting through a cross‐section of the m/g/m sandwich. The slice of epoxy is transferred to a substrate, leaving the m/g/m cross‐section exposed and facing upward. The graphene is partially etched away by oxygen plasma treatment, leaving an atomically thin trench between the two metals. Provides access to ultrathin nanogaps. Restricted to a single feature shape; non‐scalable.	^[^ ^162^ ^]^

Many challenges remain to be solved in the field of nanogap patterning, including widening the palette of patternable materials, improving control over the gap size, enhancing patterning fidelity, increasing the areal density of the nanogap features, and creating more complex array‐based structures such as 3D nanogap arrays or gradient arrays in which nanogap properties vary progressively across the width of the array. However, the techniques reviewed in this article already offer exciting opportunities to investigate and exploit nanoscale phenomena over large areas, and offer a versatile toolkit of methods for creating sub‐10 nm, sub‐5 nm, and in some cases sub‐1 nm MNGs that would have been unthinkable twenty years ago.

## Conflict of Interest

The authors declare no conflict of interest.
